# TSKS localizes to nuage in spermatids and regulates cytoplasmic elimination during spermiation

**DOI:** 10.1073/pnas.2221762120

**Published:** 2023-03-07

**Authors:** Keisuke Shimada, Soojin Park, Seiya Oura, Taichi Noda, Akane Morohoshi, Martin M. Matzuk, Masahito Ikawa

**Affiliations:** ^a^Department of Experimental Genome Research, Research Institute for Microbial Diseases, Osaka University, Suita, Osaka 565-0871, Japan; ^b^Graduate School of Medicine, Osaka University, Suita, Osaka 565-0871, Japan; ^c^Graduate School of Pharmaceutical Sciences, Osaka University, Suita, Osaka 565-0871, Japan; ^d^Center for Drug Discovery, Baylor College of Medicine, Houston, TX 77030; ^e^Department of Pathology and Immunology, Baylor College of Medicine, Houston, TX 77030; ^f^The Institute of Medical Science, The University of Tokyo, Tokyo 108-8639, Japan

**Keywords:** spermiation, spermatogenesis, male fertility, male infertility, CRISPR/Cas9

## Abstract

Spermatozoa have a streamlined shape to swim through the oviduct to fertilize oocytes. To become svelte spermatozoa, spermatid cytoplasm must be eliminated in several steps including sperm release, which is part of spermiation. Although this process has been well observed, the molecular mechanisms that underlie it remain unclear. In male germ cells, there are membraneless organelles called nuage, which are observed by electron microscopy in various forms of dense material. Reticulated body (RB) and chromatoid body remnant (CR) are two types of nuage in spermatids, but the functions of both are unknown. Using CRISPR/Cas9 technology, we deleted the entire coding sequence of testis-specific serine kinase substrate (TSKS) in mice and demonstrate that TSKS is essential for male fertility through the formation of both RB and CR, prominent sites of TSKS localization. Due to the lack of TSKS-derived nuage (TDN), the cytoplasmic contents cannot be eliminated from spermatid cytoplasm in *Tsks* knockout mice, resulting in excess residual cytoplasm with an abundance of cytoplasmic materials and inducing an apoptotic response. In addition, ectopic expression of TSKS in cells results in formation of amorphous nuage-like structures; dephosphorylation of TSKS helps to induce nuage, while phosphorylation of TSKS blocks the formation. Our results indicate that TSKS and TDN are essential for spermiation and male fertility by eliminating cytoplasmic contents from the spermatid cytoplasm.

## Significance

Spermatozoa have a streamlined shape to swim through the female reproductive tract to fertilize oocytes. However, little is known about the process of elimination of spermatid cytoplasm. When we created *Tsks*-null mice, the knockout mice cannot generate two types of nuage, reticulated body (RB) and chromatoid body remnant (CR), and are sterile with abnormal elimination of spermatid cytoplasm. Due to the absence of RB and CR, *Tsks* knockout spermatozoa have excess residual cytoplasm because the cytoplasmic contents cannot be eliminated from spermatid cytoplasm. These results suggest that TSKS-derived nuage are essential for spermatozoa to be streamlined. This study contributes to our understanding of spermiation, genetic diagnosis of idiopathic male infertility, and treatment of patients with infertility.

Spermatozoa have a structurally unique morphology compared to other cells. Each spermatozoon that forms in the testis has a streamlined morphology so it can swim through the female reproductive tract to fertilize an oocyte. To become streamlined spermatozoa, nuclear condensation (1) and cytoplasm elimination (2) are necessary for spermatids. In the process of making spermatids smaller and more streamlined, there are at least three phases as below (3). First, fluid is eliminated from the nucleus and cytoplasm during the elongation of the spermatid (2). Second, the tubulobulbar complexes eliminate spermatid cytoplasm just before sperm release (4). Third, spermatid cytoplasm is eliminated as a residual body at sperm release (5).

Spermiation is the process by which mature spermatids are released from Sertoli cells (5). After spermiation has ended, mature spermatids are released into the tubule lumen (this moment is referred to as “disengagement”), and the remnants of the spermatid cytoplasm (known as the “residual body”) are phagocytosed by Sertoli cells (6). Before disengagement, a spermatid has extensive cytoplasm around the flagellum (stage VI of the mouse seminiferous tubules) and is largely enveloped by finger-like projections of the apical Sertoli cell cytoplasm (5). As spermiation progresses, the spermatid head and flagellum gradually move farther into the tubule lumen by the lengthening of the Sertoli cell stalk (stages VII–VIII of the mouse seminiferous tubules). While the spermatid head moves up to the lumen side, its cytoplasm remains stationary within the epithelium until it is present below the level of the sperm head. This cytoplasm localized on the basal membrane side is called a spermatid “cytoplasmic lobe,” which contains organelles that are no longer used for spermatogenesis. The cytoplasmic lobe, which contains some organelles and dense materials (7), will be separated from the spermatid to become the residual body (6). Subsequently, a small amount of cytoplasm called the “cytoplasmic droplet” remains attached to the midpiece of the spermatozoon. The cytoplasmic droplet contains limited contents (8), and it is either lost during migration through the epididymis (9) or remains as a normal component of functional spermatozoa after ejaculation (10). However, excess residual cytoplasm (ERC) is often associated with dysfunctional spermatozoa and male infertility secondary to faulty spermatogenesis (11). ERC contains elevated levels of cytoplasm enzymes that produce pathological amounts of reactive oxygen species (12), which may cause oxidative stress on the spermatozoa (13). Therefore, understanding the molecular mechanisms involved in spermiation is crucial for understanding the etiology of male infertility.

In germ cells, there are membraneless organelles called nuage, which are visualized by electron microscopy as amorphous dense material during spermatogenesis (14, 15). The mechanism of nuage formation is not fully understood, but it is thought to be generated by liquid–liquid phase separation (16). Multiple materials such as 70- to 90-nm particles, satellite body, pi-body (also called intermitochondrial cement), cluster of 30-nm particles, cluster of 60- to 90-nm particles, chromatoid body (CB), chromatoid body remnant (CR), mitochondria-associated granule, reticulated body (RB), and granulated body are known to exist within the germline as nuage (15, 17). PIWI-interacting RNAs that reside in pi-body protect the gamete genome by silencing transposons, and their function has been extensively studied (18, 19). Alternatively, little is known about the roles of other nuage, such as RB and CR. RB is a dense and finely filamentous material (20), but its origin and functions are not clear. CR (also called annulus-associated CB) is a CB that migrates to the caudal pole of the nucleus of early elongating spermatids, where it forms a ring around the base of the developing flagellum (21).

Testis-specific serine kinase substrate (TSKS) was identified as a substrate for testis-specific serine kinase 1 (TSSK1) and TSSK2 (22). Although these three proteins co-localize to nuage, double knockout (KO) mice lacking TSSK1 and TSSK2 (*Tssk1/2* dKO mice) exhibit abnormal mitochondrial sheath formation (23), suggesting that these proteins and nuage are involved in mitochondrial sheath formation. However, the previous study observed abundant cytoplasm in the late spermatids at seminiferous tubule stage VIII of the *Tssk1/2* dKO mouse (23). Furthermore, an interaction between TSKS and phosphatase protein PPP1CC2 was also reported previously (24). Protein phosphatase 1 catalytic subunit gamma (*Ppp1cc*) is a member of the PP1 family of protein phosphatases that encodes two splice isoforms: the ubiquitous *Ppp1cc1* and the testis-specific *Ppp1cc2* (25). *Ppp1cc* knockout (KO) male mice also exhibit malformed mitochondrial sheath formation (26). These data indicate that the kinase and phosphatase of TSKS are involved in the formation of the mitochondrial sheath. However, the molecular mechanism of this process was not fully understood due to a lack of knowledge about *Tsks* KO mice.

In this study, we generated *Tsks* KO mice and characterized their phenotype. *Tsks* KO male mice are sterile similar to the mice lacking above proteins. Although *Tsks* KO spermatozoa have an abnormality in the midpiece similar to *Tssk1/2* dKO and *Ppp1cc* KO, we discovered that the main abnormality of *Tsks* KO is a spermiation defect. Due to the spermiation defect in *Tsks* KO testis, KO spermatozoa possess ERC, causing an apoptotic response in spermatozoa, which also causes mitochondrial sheath defects. Moreover, we revealed that TSKS is essential for formation of RB and CR, which facilitate proper spermiation.

## Results

### *Tsks* KO Male Mice Are Sterile.

**Fig. 1.**
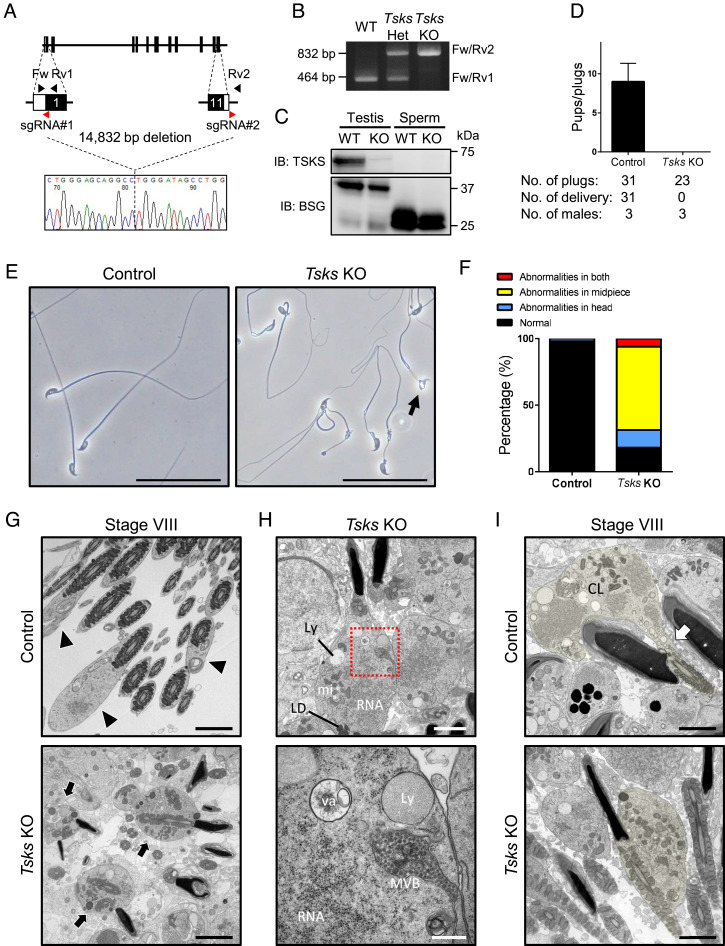
*Tsks*-deleted male mice are sterile. (*A*) KO strategy for generating *Tsks* KO mice. *Upper* panel shows diagram for *Tsks* gene. Two gRNAs (red arrowheads) were designed to target exons 1 and 11. Fw is a forward primer for genotyping; Rv1 and Rv2 are reverse primers for genotyping. *Tsks* KO mouse line that has 14,832-bp deletion was generated in the present study as *Bottom* panel. (*B*) Genotyping of *Tsks* KO-mutant mice. Fw/Rv1 and Fw/Rv2 primers in Fig. 1*A* were used. (*C*) Protein expression of TSKS in control and *Tsks* KO testis and cauda epididymal spermatozoa. Basigin was used as a loading control. basigin (BSG). (*D*) Number of litters born per plug detected. *N *= 3 males each for control, and *Tsks* KO were mated with three WT females per male. (*E*) Observation of spermatozoa obtained from the cauda epididymis. *Tsks* KO spermatozoa have abnormalities in the midpiece and/or head shape. Arrow indicates abnormal head shape. (Scale bar, 50 μm.) (*F*) Graph indicates frequencies of abnormalities in control and *Tsks* KO spermatozoa (*N *= 3, a hundred spermatozoa were counted from each animal). (*G*) Ultrastructural images of spermatozoa in the seminiferous tubule lumen at stage VIII. The spermatozoa in the control testis have tiny contents inside their cytoplasmic droplets (arrowheads), but the ones in *Tsks* KO testis have an abundance of contents inside their cytoplasm (arrows). (Scale bar, 2 μm.) (*H*) Ultrastructural images of spermatozoa after spermiation in *Tsks* KO testis. *Lower* panel shows magnified images of the boxed areas. (Scale bars, 2 μm [*Upper*] and 500 nm [*Lower*].) LD, lipid droplet; mi, mitochondria; MVB, multiple vesicular bodies Ly, lysosome; va, vacuole. (*I*) Ultrastructural images of spermiation. The cytoplasmic lobe (CL) of control spermatozoa contains RNA, mitochondria, lipid droplets, vacuoles, and multiple vesicular bodies, which are transported from the sperm cytoplasm and included in the residual bodies. Arrow indicates spermatid stalk. There was no spermatid stalk in *Tsks* KO spermatids, and the contents that should be in the cytoplasmic lobe were located near the flagellum. The cytoplasm of the center spermatid is pseudocolored in yellow for clarity. (Scale bar, 2 μm.)

To confirm testis-specific expression of *Tsks* (22, 27), we performed RT-PCR using multiple tissues from adult mice. RT-PCR revealed that *Tsks* is expressed in the testis, but not in other tissues, consistent with previous studies (*SI Appendix*, Fig. S1*A*) (27). To reveal the functions of TSKS in male fertility, we generated *Tsks* KO mice. *Tsks* KO mice were successfully generated by the CRISPR/Cas9 system using embryonic stem (ES) cells (28). Exons 1 to 11 were deleted (a 14,832–base pair deletion), as demonstrated by genomic DNA sequencing and PCR (Fig. 1 *A* and *B*). To confirm ablation of the TSKS protein since we had deleted all of the coding exons of the *Tsks* gene, we generated a polyclonal antibody against TSKS. Western blot analysis revealed that TSKS protein was present in the testis of control but absent in KO testis (Fig. 1*C*). We also found that TSKS was detected in the testis but not in spermatozoa. When *Tsks* KO male mice were mated with wild type (WT) females, no pups were produced (Fig. 1*D*), indicating that *Tsks* KO male mice are sterile.

To understand the cause of the sterility, we checked the sperm morphology. We found that over 80% of *Tsks* KO spermatozoa have abnormalities in the midpiece and/or head shape (Fig. 1 *E* and *F*). Scanning electron microscope (SEM) analysis revealed that *Tsks* KO spermatozoa have abnormal mitochondrial loss in the midpiece and abnormal head shape (*SI Appendix*, Fig. S1*B*), consistent with the light microscopic findings.

When we measured the sperm motility parameters of *Tsks* KO spermatozoa using computer-assisted sperm analysis, both sperm motility and progressive sperm rate were significantly lower than those of control (*SI Appendix*, Fig. S1 *C* and *D*). To test the fertilizing ability of *Tsks* KO spermatozoa, we performed in vitro fertilization and found that the KO spermatozoa failed to fertilize well oocytes in all conditions (*SI Appendix*, Fig. S1*E*). Then, we determined the viability of *Tsks* KO spermatozoa by propidium iodide staining (*SI Appendix*, Fig. S1*F*). The live sperm rate of *Tsks* KO spermatozoa was comparable with that of control (*SI Appendix*, Fig. S1*G*), suggesting that sperm death was not the cause of the extremely low motility of *Tsks* KO spermatozoa. To investigate if *Tsks* KO sperm nuclei can produce viable pups despite impaired sperm morphology, we conducted intracytoplasmic sperm injection (ICSI). Oocytes injected with *Tsks* KO spermatozoa developed into 2-cell embryos, and the embryos were transplanted into pseudopregnant mothers (*SI Appendix*, Fig. S1*H*). Four heterozygous pups were obtained (*SI Appendix*, Fig. S1 *I* and *J*) and grew normally. These results indicate that *Tsks* KO sperm nuclei could activate eggs and produce viable pups, and the infertility phenotype observed in *Tsks* KO males could be rescued with ICSI.

To determine the cause of the abnormal sperm morphology, we examined the testis. Both gross morphology and testicular weight of the KO mice were not significantly different from those of WT mice (*SI Appendix*, Fig. S2 *A* and *B*). Periodic acid-Schiff staining showed that many mature sperm heads were abnormally observed in the stage IX seminiferous tubules of *Tsks* KO testis (*SI Appendix*, Fig. S2*C*), the same as in *Tssk1/2* dKO testis (23). In addition, *Tsks* KO testis appears to be crowded with a large number of sperm heads present in the lumen of stage VIII seminiferous tubules (*SI Appendix*, Fig. S2*D*). As spermatozoa in the control testis at the same stage are in a row in the lumen of the testes, spermiation defects were suggested in *Tsks* KO mouse testes.

To study the spermiation defects in more detail, transmission electron microscopy (TEM) was used to examine the ultrastructure of the spermatozoon. TEM analysis revealed that *Tsks* KO spermatozoa have normal axonemes (*SI Appendix*, Fig. S2*E*). After their release from Sertoli cells, control spermatozoa contain cytoplasmic droplets with limited numbers of organelles and other structures (Fig. 1*G*, arrowheads). In contrast, *Tsks* KO spermatozoa have an abundance of contents inside their cytoplasm near the midpiece (Fig. 1*G*, arrows). The cytoplasm of *Tsks* KO spermatozoa contains RNA, mitochondria, vacuoles, lipid droplets, lysosomes, and multiple vesicular bodies (Fig. 1*H*), which normally should be separated from the sperm cytoplasm as residual bodies. Toluidine blue, which could be used for residual body staining (29), stained these cytoplasmic spots dark blue in the *Tsks* KO lumens of seminiferous tubules around stages VII-VIII (*SI Appendix*, Fig. S2*F*, arrowheads). These results suggest that *Tsks* KO spermatozoa contain ERC that should be separated during spermiation. When we observed the moment of spermiation, a thin spermatid stalk that connects spermatids and the cytoplasmic lobe was observed in control testes; however, these were not observed in *Tsks* KO seminiferous tubules at stage VIII (Fig. 1*I*). When we observed spermatozoa in the cauda epididymis with TEM, *Tsks* KO spermatozoa with ERC have incomplete membrane and/or substances with high electron density (*SI Appendix*, Fig. S3*A*). In contrast, the portion of *Tsks* KO spermatozoa without ERC showed few abnormalities (*SI Appendix*, Fig. S3*A*, arrows). Using an apoptosis marker [cleaved (active) caspase-3], we observed that *Tsks* KO spermatozoa in the epididymis expressed cleaved caspase-3, indicating that the spermatozoa underwent apoptosis (*SI Appendix*, Fig. S3*B*). In summary, *Tsks* KO spermatozoa demonstrate ERC (Fig. 1*G*), structural defects in the midpiece (Fig. 1*E*), low sperm motility (*SI Appendix*, Fig. S1 *C* and *D*), and increased apoptosis (*SI Appendix*, Fig. S3*B*). Together, these defects underlie the sterility of the *Tsks* KO males.

### TSKS Is Localized to Two Different Nuage, Reticulated Body and Chromatoid Body Remnant, in Spermatids.

**Fig. 2.**
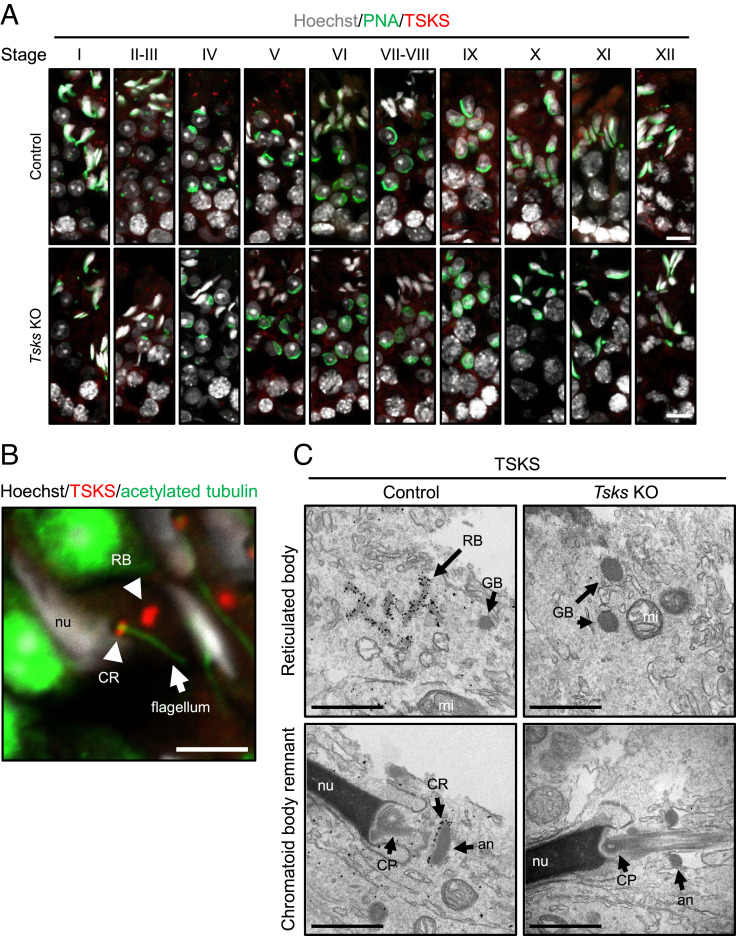
TSKS localized on reticulated body and chromatoid body remnant. (*A*) Immunofluorescence analysis of stage I–XII seminiferous tubules in the testis of control and *Tsks* KO mice. Spermatogenic stages were identified by the morphology of the nucleus and acrosome stained with Hoechst 33342 (white) and PNA lectin (green), respectively. TSKS was detected with antibodies to TSKS (red). While TSKS expresses step 11 to 15 spermatids in the control testis, TSKS does not express in *Tsks* KO testis. (Scale bar, 10 μm.) (*B*) Immunostaining of TSKS (red) in WT mouse testis. Hoechst 33342 (white) and acetylated tubulin (green) were used to visualize the nuclei and tubulin, respectively. (Scale bar, 5 μm.) (*C*) Detection of immunolabeled TSKS in the testis by TEM using anti-TSKS antibody incubated with 1.4-nm gold particle–conjugated secondary antibody. TSKS localizes on two different nuage: RB and CR in WT testis, but those nuage were disappeared in *Tsks* KO testis. (Scale bar, 1 μm.) RB, reticulated body; CR, chromatoid body remnant; GB, granulated body; CP, connecting piece; mi, mitochondria; nu, nuclear; an, annulus.

Previous studies revealed that TSKS shows intracytoplasmic localization in elongating spermatids (22, 23). To confirm this in more detail, we performed immunofluorescent analysis using a newly developed anti-TSKS antibody. Specific TSKS protein expression is observed in step 11 to 15 spermatids in the control testis but absent in the KO (Fig. 2*A*). At a higher magnification, TSKS localizes to the anterior end of the axoneme and to nondescript cytoplasm (Fig. 2*B*) as previously described (23). The previous study has referred to these structures as “CB ring and satellite,” which is well described by Fawcett et al. (21). To further confirm TSKS localization in these structures, we performed immunoelectron microscopy (immuno-EM) using gold-labeled antibodies. Gold particles were found in germ cell RB and CR of ultrathin testis sections from control mice stained with an antibody against TSKS, whereas these particles and organelles were absent in the *Tsks* KO testis (Fig. 2*C*). These results directly indicate that TSKS localizes in both RB and CR but not chromatoid satellite, and TSKS is essential for RB and CR formation. Both RB and CR are membraneless organelles called nuage, which are composed of electron-dense materials (17). Previous studies reported that RB is present in spermatids from step 14 to 16 spermatids in rat (corresponding to step 12 to 14 spermatids in mouse) and disappeared soon after (20). Another report showed that CR is associated with the annulus during its caudal migration to the annulus (21). While there have been no previous reports for the functions of RB and CR, our present study suggests that RB and CR play important roles in spermiation.

### TSKS Interacts with TSSK1, TSSK2, and PPP1CC2 in the Testis.

Previous studies have reported that TSKS interacts with TSSK1, TSSK2, and PPP1CC2 (22, 24). We confirmed these interactions by western blot analysis of the immunoprecipitants and found that TSKS interacts with TSSK1, TSSK2, and PPP1CC2 but not with PPP1CC1 (*SI Appendix*, Fig. S4*A*). We therefore tested for the expression levels of these interactomes in *Tsks* KO testis and spermatozoa and found that all expression levels were decreased in the testis or spermatozoa (*SI Appendix*, Fig. S4*B*). We then performed immunofluorescent analysis using anti-TSSK1 and TSSK2 antibodies. Both TSSK1 and TSSK2 were expressed with strong signals in step 11-15 spermatids at stage V seminiferous tubules in control similar to the expression of TSKS. However, both TSSK1 and TSSK2 are expressed throughout the cytoplasm in elongated spermatids after TSKS disappeared (stage VI to VIII seminiferous tubules). In contrast, no specific signals for TSSK1 and TSSK2 are observed in *Tsks* KO testis similar to TSKS (*SI Appendix*, Fig. S4 *C* and *D*). Coimmunofluorescent staining for TSKS and TSSK2 (*SI Appendix*, Fig. S4*E*) and TSSK1 and TSSK2 (*SI Appendix*, Fig. S4*F*) indicates that these three proteins co-localize in elongating spermatids. Immuno-EM also revealed that TSSK2 localizes in RB and CR similar to TSKS, but TSSK2 is not detected in *Tsks* KO spermatids (*SI Appendix*, Fig. S4*G*).

We subsequently checked PPP1CC2 localization and showed that PPP1CC2 is expressed in the cytoplasm of secondary spermatocytes, spermatids, and spermatozoa (*SI Appendix*, Fig. S5*A*) as previously described (26). The PPP1CC2 expression pattern is not changed in *Tsks* KO testis in contrast to the TSSK1 and TSSK2 findings. Although it is not mentioned in the previous study, prominent expression of PPP1CC2 is observed in step 14 and initial step 15 spermatids (*SI Appendix*, Fig. S5*A*). When we performed coimmunofluorescent staining for TSSK2 and PPP1CC2, these proteins are closely located near the neck region of elongating spermatids but do not co-localize (*SI Appendix*, Fig. S5*B*).

### TSKS Interacts with Cytoplasmic Protein HSPA1L.

Because TSKS-derived nuage (TDN), RB and CR, disappear prior to spermiation, TSKS is unlikely to directly affect spermiation. Therefore, it is possible that TSKS is indirectly related to spermiation due to some alterations caused by the loss of TDN. To elucidate the causes of spermiation defects, we determined the interactomes of TSKS in addition to the above-described kinases and phosphatase. TSKS protein complexes were isolated from testis lysates of control and *Tsks* KO mice and subjected to mass spectrometry (MS) analysis. TSKS, TSSK1, TSSK2, PPP1CC, ODF1, ACTB, and several heat shock proteins were detected from immunoprecipitates (*SI Appendix*, Fig. S6*A*). We confirmed their interactions with TSKS by immunoblot analysis after immunoprecipitation. TSKS interacts with HSPA1L, HAPA1, and ACTB but not ODF1 and HSPA8 (*SI Appendix*, Fig. S6*B*). HSPA1L and HSPA1 expression levels did not change in *Tsks* KO testis and spermatozoa unlike TSKS kinases and phosphatase (*SI Appendix*, Fig. S6*C*). *Hspa1l* is a member of the 70-kDa heat shock protein family gene and shows testis-enriched expression, but its functions are unclear (30). HSPA1L starts to express from step 12 spermatids as previously described (30). HSPA1L is initially located in the spermatid cytoplasm in the lumen of seminiferous tubules at stage IV of the spermatogenic cycle and subsequently relocalizes to near the sperm head (stage VI) (*SI Appendix*, Fig. S6*D*). In stage VIII seminiferous tubules, HSPA1L localizes to the cytoplasmic lobe and around the midpiece (*SI Appendix*, Fig. S6 *D* and *E*). After spermiation (stage IX), HSPA1L is observed in the residual bodies (*SI Appendix*, Fig. S6 *D* and *E*). However, in *Tsks* KO mice, HSPA1L at stage VIII seminiferous tubules was also observed in the spermatid cytoplasm in the lumen. In addition, HSPA1L was expressed in cytoplasmic lobes, residual bodies, and around the midpiece at stage IX seminiferous tubules (*SI Appendix*, Fig. S6*F*). Therefore, spermiation defects observed in *Tsks* KO testis might be related to abnormal HSPA1L localization. TSKS also interacts with HSPA1, a paralog of HSPA1L (*SI Appendix*, Fig. S6*B*). Amino acid sequence of HSPA1L is similar to that of HSPA1 (HSPA1A and HSPA1B) (*SI Appendix*, Fig. S7*A*). We also found that HSPA1 is expressed in the cytoplasmic lobes and residual bodies similar to HSPA1L (*SI Appendix*, Fig. S7*B*). These results suggest that TSKS-interacting heat shock proteins, HSPA1 and HSPA1L, are related to spermiation, although the functions of these heat shock proteins are still unknown.

### Expression of TSKS Causes Nuage to Appear in Cultured Cells.

**Fig. 3.**
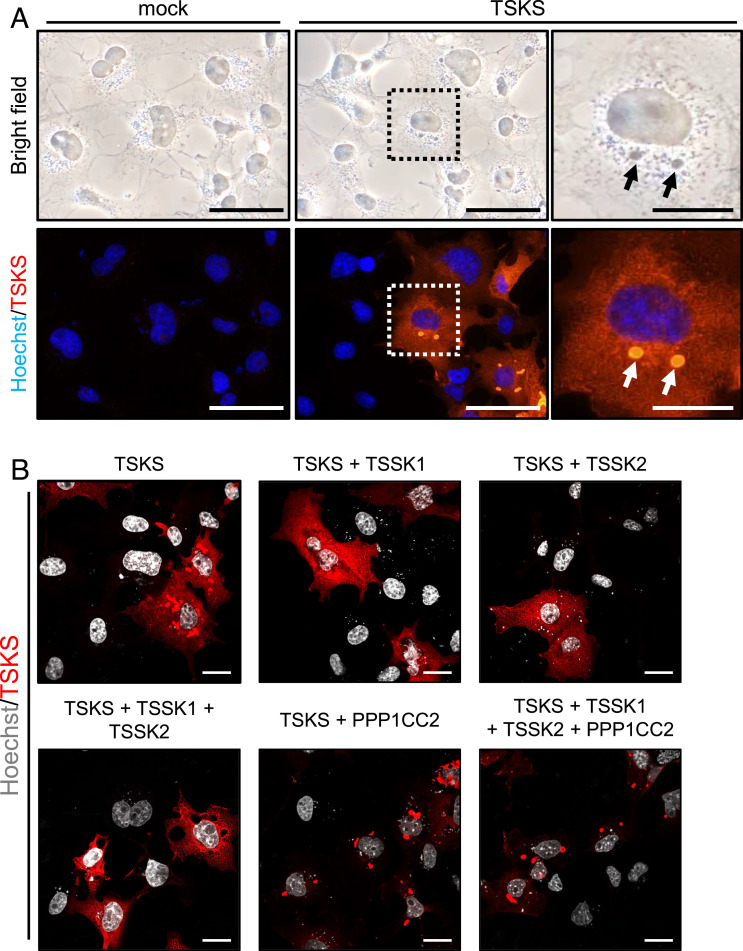
TSKS expression induces amorphous droplets in the cell. (*A*) Bright-field images (*Upper*) and fluorescence images (*Lower*) after transient expression of TSKS in COS-7 cells. COS-7 cells were transiently expressed with mock vector or 1D4-tagged TSKS expression vector and stained with 1D4 (red) and Hoechst 33342 (blue) to visualize TSKS and nuclei, respectively. The cells were observed using phase contrast fluorescence microscopy. *Right*-most panels show magnified images of the boxed areas. Arrows indicate droplets which highly express TSKS. (Scale bars, 50 μm [*Left* and *Center*] and 20 μm [*Right*].) (*B*) COS-7 cells transiently expressed with TSKS expression vector and/or its kinase, and phosphatase expressing vectors were stained with TSKS (red). Hoechst 33342 (white) was used to visualize the nuclei. Cells expressing TSKS alone and TSKS plus PPP1CC2 induced droplets. (Scale bar, 20 μm.)

To further reveal the molecular function of TSKS, we generated a *Tsks* expression vector with a 1D4 tag sequence inserted at the C terminus. When we introduced the vector into COS-7 cells, amorphous “droplets” appeared in the cytoplasm of *Tsks*-expressing cells. TSKS is expressed diffusely throughout the cytoplasm and is strongly expressed in the cytoplasmic droplets (Fig. 3*A*), indicating that TSKS is a major component of the droplets. We then hypothesized that phosphorylation and dephosphorylation of TSKS by TSSK1, TSSK2, and PPP1CC2 may be important for TSKS functions. To test the hypothesis, we generated expression vectors for *Tssk1*, *Tssk2,* and *Ppp1cc2* with epitope tags. *Tsks* expression vector and its kinase and/or phosphatase gene expression vectors were co-transfected into HEK293T cells by transient transfection, and cell lysates were examined by immunoblot analysis. As anticipated, the size of the TSKS band differs depending on the presence or absence of TSSK1/TSSK2 kinase or PPP1CC2 phosphatase (*SI Appendix*, Fig. S8*A*). Using Phos-tag gels, which allow the separation of phosphorylated forms of a protein (31), phosphorylation levels of TSKS are increased with co-transfection with TSSK1 and TSSK2 and reduced in the presence of PPP1CC2 (*SI Appendix*, Fig. S8*B*), indicating that TSKS is a cellular substrate of TSSK1, TSSK2, and PPP1CC2. We also found that the phosphorylation ability of TSSK2 is higher than that of TSSK1, and the dephosphorylation ability of PPP1CC2 is stronger than the phosphorylation ability of TSSK1 and TSSK2. In contrast to TSKS, transiently expressed TSSK1 and TSSK2 are diffusely detected in the cytoplasm, while PPP1CC2 is observed in both the nucleus and cytoplasm, and none of the proteins induced droplets (*SI Appendix*, Fig. S8*C*). To determine whether phosphorylation levels of TSKS affect its functions, we co-expressed TSKS with kinase and/or phosphatase proteins in COS-7 cells. TSKS expression spread throughout the cytoplasm when kinase protein was co-expressed with TSKS. However, these changes were recovered by the existence of PPP1CC2 (Fig. 3*B*), indicating that dephosphorylated TSKS generates droplets in the cultured cells.

**Fig. 4.**
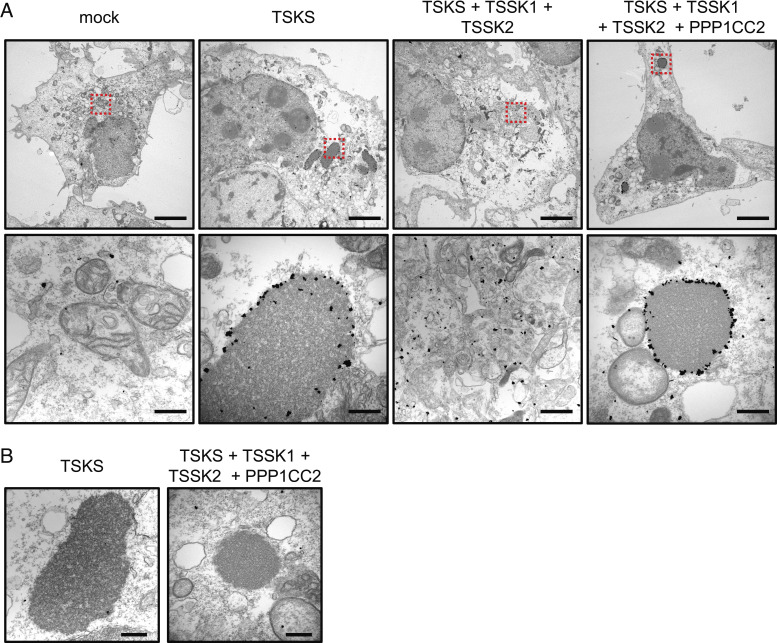
Expression of TSKS induces nuage. (*A*) COS-7 cells were transiently expressed with mock vector or 1D4-tagged TSKS expression vector or TSKS and its kinase and phosphatase expression vectors. Immunolabeled 1D4-tagged TSKS in COS-7 cells was detected by TEM using anti-1D4 antibody incubated with 1.4 nm gold particle–conjugated secondary antibody. *Bottom* panels show magnified images of the boxed areas. TSKS localized on the edge of nuage. (Scale bars, 5 μm [*Upper*] and 500 nm [*Lower*].) (*B*) Magnified images of nuage observed in COS-7 cells expressing TSKS alone and TSKS with its kinase and phosphatase. TSKS-derived nuage have high electron density. (Scale bar, 500 nm.)

To reveal the identity of the TSKS-positive droplets, we performed immuno-EM using gold-labeled antibodies. Immuno-EM revealed that TSKS localized on the surface of nuage-like structures which are not observed in the mock-transfected COS-7 cells lacking expression of TSKS (Fig. 4*A*). This result indicates that amorphous droplets caused by the expression of TSKS show nuage-like structures. The nuage-like structures are absent in the COS-7 cells after co-transfection of *Tsks*, *Tssk1*, and *Tssk2* but return in the *Tsks*, *Tssk1*, *Tssk2,* and *Ppp1cc2* quadruple transfection (Fig. 4*A*). These results are coincident with immunohistochemical staining (Fig. 3*B*) and indicate that TSKS without phosphorylation induces nuage-like structures in cultured cells. These nuage-like structures are amorphous materials with no limiting membrane that is the feature of nuage (Fig. 4*B*). But the nuage-like structures differ in morphology from RB and CR in vivo (Fig. 2*C* and *SI Appendix*, Fig. S4*G*), and their morphology is not threadlike (Fig. 4*B*). These results suggest that TSKS is essential for both RB and CR formation, although additional proteins are necessary for proper formation of RB andCR.

## Discussion

**Fig. 5.**
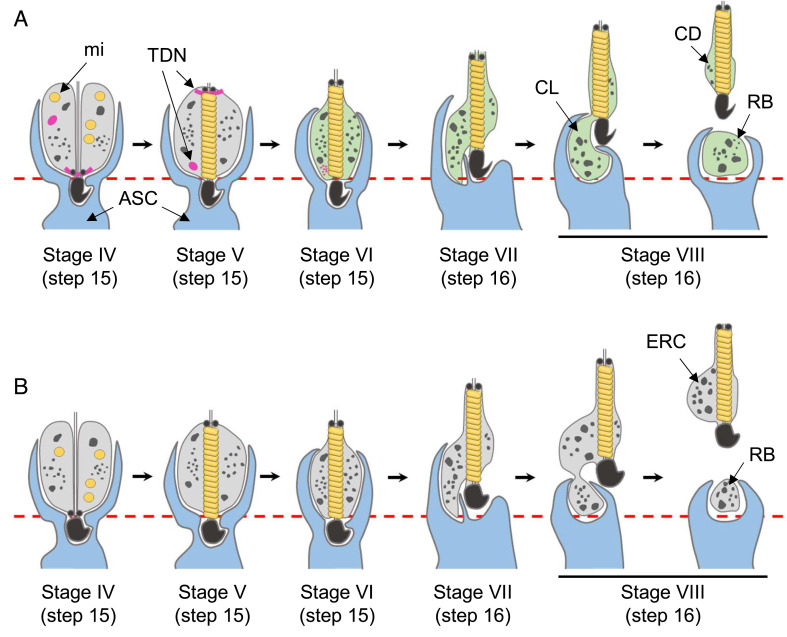
TSKS functions in spermiation. (*A* and *B*) Schematic models for spermiation in WT (*A*) and *Tsks* KO mice (*B*). (*A*) Spermatid cytoplasm is eliminated by apical Sertoli cell cytoplasm in WT testis. TSKS and TDN are present in step 15 spermatid cytoplasm until stage V seminiferous tubules. However, TSKS and TDN disappear at stage VI, which allows TSSK1 and TSSK2 to localize throughout the cytoplasm (light green). (*B*) *Tsks* KO spermatozoa possess ERC due to spermiation defects caused by the absence of TDN. mi, mitochondria; TDN, TSKS-derived nuage; ASC, apical Sertoli cell; CL, cytoplasmic lobe; CD, cytoplasmic droplet; RB, residual body; ERC, excess residual cytoplasm.

In the present study, we generated *Tsks* KO mice using the CRISPR/Cas9 system. Because of the abnormal formation of ERC in *Tsks* KO spermatozoa (Fig. 1*G*), KO male mice are infertile (Fig. 1*D*). Since ERC is formed in *Tsks* KO spermatozoa because of spermiation defects, we will now summarize the spermiation process and TSKS behavior. In normal spermiogenesis in mice, TSKS emerges on RB and CR (TDN) from step 11 spermatids and maintains expression on TDN until step 15 (Fig. 2 *A* and *C*). Spermatids that have just progressed to step 15 have an abundance of cytoplasm (Fig. 5*A*, Stage IV). The caudal migration of both the annulus and the associated CR is observed at middle step 15 spermatids (21, 32). After the migration, mitochondria align around the axoneme (Fig. 5*A*, Stage V) (33). During these processes, the cytoplasm of spermatids has been largely enveloped by finger-like projections of the apical Sertoli cell cytoplasm. As spermatids progress through late step 15, TSKS and TDN disappear, and TSSK1 and TSSK2 disperse throughout the cytoplasm (Fig. 5*A*, Stage VI). Once spermiogenesis has progressed to step 16, the spermatid head and flagellum move to the lumen side. However, spermatid cytoplasm and Sertoli cell cytoplasm remain stationary, enabling sperm cytoplasm to flow to the basal membrane side (Fig. 5*A*, Stage VII) (5). Then, the volume of cytoplasm around the sperm flagellum is reduced, and the protruded cytoplasm is referred to as the cytoplasmic lobe (Fig. 5*A*, Stage VIII, *Left*). As a result of breakage of the spermatid stalk, the cytoplasmic lobe is separated from the spermatid (disengagement). After disengagement, the cytoplasmic lobe becomes a residual body and released spermatozoa with cytoplasmic droplets migrate into the epididymis (Fig. 5*A*, Stage VIII, *Right*).

Alternatively, TSKS deletion hampers spermiation. While the majority of the cytoplasmic lobes of *Tsks* KO spermatids have been enveloped by Sertoli cells (*SI Appendix*, Fig. S2*F*), KO spermatozoa immediately after disengagement possess ERC that contains an abundance of contents (Fig. 1 *G* and *H*). This indicates that transportation of the cytoplasmic contents to the cytoplasmic lobe and/or envelopment of the cytoplasmic lobe by Sertoli cells are insufficient (Fig. 5*B*, Stage VIII, *Left*). Because of these abnormalities, *Tsks* KO spermatids could not disengage their cytoplasm appropriately (Fig. 1*I*) and have an abundance of contents within their cytoplasms (Fig. 5*B*, Stage VIII, *Right*). As assumed in previous studies (13), oxidative stress characterized by mitochondrial damage associated with peroxidative damage to the sperm plasma membrane (34) is thought to be induced in *Tsks* KO spermatozoa (*SI Appendix*, Fig. S3 *A* and *B*) due to ERC. Because of the disruption of the sperm plasma membrane (*SI Appendix*, Fig. S3*A*), it was difficult to detect ERC in *Tsks* KO spermatozoa collected from the cauda epididymis, although mitochondrial abnormalities were observed by optical microscopy (Fig. 1*E*). A previous study revealed that *Tssk1/2* dKO male mice lacked significant expression of TSKS in spermatids. These dKO spermatids also possessed a collapsed mitochondrial sheath and had an abundance of cytoplasm during spermiation (23). Therefore, the defect observed in *Tssk1/2* dKO is thought to be the same one that is observed in *Tsks* KO male mice. Mitochondrial sheath defects observed in *Ppp1cc* KO mice (26) might be the same mechanism as in *Tsks* KO.

Although spermiation defects caused by tubulobulbar complexes or apical ectoplasmic specialization malfunction have been reported (35–38), defects caused at disengagement are not well reported. *Spem1* KO male mice are infertile due to aberrant cytoplasm removal (39). Although *Spem1* KO spermatozoa possess ERC in the head and neck region, the cytoplasmic contents are totally different from *Tsks* KO spermatozoa. *Spem1* KO epididymal spermatozoa have membranous vacuoles inside the cytoplasm but do not have mitochondria, lysosomes, and multiple vesicular bodies observed in the cytoplasm of the *Tsks* KO. In addition, *Spem1* KO epididymal spermatozoa have intact cytoplasmic membranes without apoptosis induction different from *Tsks* KO. Therefore, the differences between *Tsks* KO and *Spem1* KO spermatozoa suggest that TSKS and TDN function to transport cytoplasmic contents from spermatid cytoplasm to cytoplasmic lobes.

In the present study, we also found that *Tsks* KO spermatozoa underwent apoptosis (*SI Appendix*, Fig. S3*B*). Despite this, the live sperm rate of *Tsks* KO spermatozoa was comparable to that of control spermatozoa (*SI Appendix*, Fig. S1*G*), and pups obtained by ICSI from *Tsks* KO spermatozoa grew normally (*SI Appendix*, Fig. S1 *H*–*J*). The lack of abnormalities in the live sperm rate and fertilization ability was thought to be due to the isolation of the sperm heads from the abnormal cytoplasmic components in ERC (Fig. 1 *G* and *I*).

Although we showed that TSKS and TDN function during elimination of cytoplasmic contents from spermatids, we were unable to elucidate the molecular mechanisms involved in the elimination process. We revealed that TSKS induces nuage formation in vitro (Fig. 3*A*), but the morphology of the nuage was totally different from RB and CR (Fig. 2*C*). This indicates that TSKS is essential for both RB and CR formation but is not sufficient. TSKS phosphorylation is crucial for nuage formation because phosphorylated TSKS does not form nuage and expresses whole cytoplasm, while dephosphorylated TSKS appears only in nuage (Fig. 3*B*). Even though there are many missing links between TSKS function and the spermiation process, it may be possible to understand these mechanisms if we acquire more knowledge about TSKS and related proteins.

The present study also uncovered multiple heat shock proteins from immunoprecipitates using TSKS antibody (*SI Appendix*, Fig. S6*A*), and HSPA1L is expressed in spermatid cytoplasm from step 12, cytoplasmic lobe and residual body (*SI Appendix*, Fig. S6 *D* and *E*) as HSPA2 (40). Therefore, heat shock proteins such as HSPA1L and HSPA2 are strongly suspected to be involved in spermiation. However, we were unable to determine how HSPA1L and HSPA2 (and other heat shock proteins) are involved in spermiation. Because defects in spermiation cause ERC, which induces male infertility in humans (13), understanding of the molecular mechanism of spermiation can lead to the development of fertility treatments. In contrast, the findings may lead to the development of male contraceptives if we could find compounds that inhibit spermiation without dramatically disrupting testis size. Our studies can contribute to our understanding of spermiation, genetic diagnosis of idiopathic male infertility, and treatments of patients with infertility.

## Materials and Methods

Additional information is provided in **SI Appendix*, Materials and Methods*.

### Animals.

All animal experiments were approved by the Animal Care and Use Committee of the Research Institute for Microbial Diseases, Osaka University (Osaka, Japan), in accordance with the animal testing guidelines and regulations. Animals were housed in a temperature-controlled environment with 12-h light cycles and free access to food and water. B6D2F1 (C57BL/6 × DBA2), ICR, or C57BL/N mice were used as embryo donors, foster mothers, or gene cloning, respectively. These mice were purchased from CLEA Japan, Inc. or Japan SLC, Inc.

### Generation of Knockout Mouse Using the CRISPR/Cas9 System.

*Tsks* KO mice were produced with the CRISPR/Cas9 genome editing system. To avoid the off-target editing, CRISPRdirect software (https://crispr.dbcls.jp/) was used (41). ES cells were used to produce the mice, as previously described (28). We designed guide RNAs and inserted the sequence into the pX459 V2.0 plasmid (#62988, Addgene). The EGR-G01 ES cells were co-transfected with two guide RNA–inserted vectors using Lipofectamine LTX with Plus Reagent (ThermoFisher Scientific). Cells were selected using puromycin and genotyping. Mutant ES clones with normal karyotypes were aggregated into 8-cell or morula stage ICR embryos, and they were cultured to the blastocyst stage. A pseudopregnant female ICR recipient was used to implant them into the uterus 2.5 d after mating with a vasectomized male. The resulting chimeric male spermatozoa were used for intracytoplasmic sperm injection (ICSI) to obtain KO mice. Genotyping was conducted by Sanger sequencing and PCR. The primers and PCR conditions for genotyping are listed in *SI Appendix*, Table S1.

### Electron Microscopy.

Scanning electron microscope (SEM) analysis of spermatozoa was performed as previously described (42).

TEM analysis of the testis, spermatozoa, and COS-7 cells was performed as previously described (42, 43). For immunoelectron microscopy, samples were incubated with rabbit anti-TSKS antibody, mouse anti-TSSK2, or rabbit anti-1D4 antibody; slides were washed to remove the primary antibody, and the tissue sections were incubated with goat anti-rabbit or anti-mouse IgG coupled to 1.4-nm gold (Nanogold, Nanoprobes). Immunogold-labeled TSKS and TSSK2 in the testis and TSKS in COS-7 cells were examined using a JEM-1400 plus electron microscope (JEOL) at 80 kV with a CCD Velta 2K × 2K camera (Olympus).

### Immunofluorescence.

Immunofluorescence analysis of testes was performed using cryosections as previously described (44) with slight modification. Testes were fixed with 4% paraformaldehyde (PFA) at 4 °C for 1 h and transferred sequentially into 10%, 15%, and 20% sucrose in phosphate-buffered saline (PBS). Fixed testes were embedded in OCT compound (Sakura Finetek), and 10-μm sections were prepared with a cryostat (CryoStar NX70, ThermoFisher Scientific). Antigen retrieval was performed in citric acid buffer (pH 6.0) at 95 °C for 20 min. After washing, the samples were permeabilized with 0.1% Triton X-100 (Nacalai Tesque) in PBS for 15 min, washed again, and then blocked with 3% bovine serum albumin (BSA, Merck) for 30 min. The sections were incubated overnight at 4 °C with primary antibodies in 3% BSA. After three times washing with PBS, the appropriate Alexa Fluor–conjugated secondary antibodies (ThermoFisher Scientific) and Alexa Fluor–conjugated lectin PNA (ThermoFisher Scientific) were added to the slides and incubated for 2 h at room temperature. After three times washing, the sections were stained with Hoechst 33342 (ThermoFisher Scientific) for visualizing nuclei. After washing three times, the sections were coverslipped with Immu-Mount (ThermoFisher Scientific).

Immunofluorescence analysis of cultured cells was performed as previously described with slight modification (42). COS-7 cells (1.5 × 10^5^ cells) were seeded on coverslips in a 6-well plate. After 6 to 8 h, expressing vectors were transiently transfected into COS-7 cells using PEI MAX (Polysciences). After 40 h, cells were fixed by 4% PFA and permeabilized with 0.5% Triton X-100. Cells were blocked with 3% BSA and immunostained. The cells were incubated with primary antibodies overnight at 4 °C. After washing three times, the cells were incubated with Alexa Fluor–conjugated secondary antibodies for 2 h at room temperature. The cells were then washed three times and stained with Hoechst 33342 for visualizing nuclei. After washing three times, the cells were mounted on MAS-coated glass slides (Matsunami) with Immu-Mount.

Microscopic images were obtained using a Nikon Eclipse Ti microscope connected to a C2 confocal module. Fluorescent images were false-colored and cropped using ImageJ software (version 2.0.0, NIH). The antibodies used in this study are listed in *SI Appendix*, Table S2.

### Statistical Analysis.

Statistical analyses were performed using a two-tailed unpaired *t* test (n ≥ 3) by GraphPad Prism 6 (GraphPad). *P* values less than 0.05 were considered significant. Data represent the means, and error bars indicate SD.

## Supplementary Material

Appendix 01 (PDF)

## Data Availability

The *Tsks* KO mouse strain used in this study was deposited under the name STOCK *Tsks^em1Osb^*, and available through either the Riken BioResource Center (Riken BRC; Tsukuba, Japan, https://mus.brc.riken.jp/en/search_for_mouse_strain) or the Center for Animal Resources and Development, Kumamoto University (CARD; Kumamoto, Japan, https://cardmice.com/rbase/changelang?lang=en). The stock ID number of *Tsks* KO mouse strain is 11062 (Riken BRC) or 2969 (CARD), respectively. All study data are included in the manuscript and/or *SI Appendix*.
