# Supplementary material for: TSKS localizes to nuage in spermatids and regulates cytoplasmic elimination during spermiation
Source: Proc Natl Acad Sci U S A. 2023 Mar 7;120(11):e2221762120. doi: 10.1073/pnas.2221762120 (PMC10242716; doi:10.1073/pnas.2221762120)
Supplement: Supplementary file 1 — Appendix 01 (PDF) [file pnas.2221762120.sapp.pdf]

Supporting Information for

TSKS localizes to nuage in spermatids and regulates cytoplasmic elimination during spermiation

Keisuke Shimada, Soojin Park, Seiya Oura, Taichi Noda, Akane Morohoshi, Martin M. Matzuk, and Masahito Ikawa

Corresponding author: Keisuke Shimada, Martin M. Matzuk and Masahito Ikawa
Email:
K.S. shimada-k@biken.osaka-u.ac.jp,
M.M.M. mmatzuk@bcm.edu,
M. I. ikawa@biken.osaka-u.ac.jp

This PDF file includes:

SI Materials and Methods
Figures S1 to S8
Tables S1 to S2
SI References

SI Appendix

SI Materials and Methods

RT-PCR

RT-PCR was conducted as previously described (1). RNA was prepared from multiple adult tissues of C57BL/6N mice using TRIzol (ThermoFisher Scientific, Waltham, MA, USA) according to the manufacturer's protocol. The obtained RNA was reverse transcribed to complementary DNA (cDNA) with the SuperScript IV Reverse Transcriptase (ThermoFisher Scientific) using an oligo (dT) primer. PCR was then performed using 10 ng cDNA with the primers listed in Table S1. The amplification conditions for the subsequent PCR were 5 min at 94 °C, followed by 35 cycles of 94 °C for 30 s, 60 °C for 30 s, and 72 °C for 30 s, with a final 7 min extension at 72 °C.

Immunoblot analysis

Immunoblot analysis was performed as previously described (2). Cell lysates from HEK293T cells were obtained using a Pierce IP Lysis Buffer (ThermoFisher Scientific), and proteins from testis or spermatozoa were extracted using a T-PER (tissue protein extraction reagent, ThermoFisher Scientific). Proteins were separated by SDS-PAGE under reducing conditions and transferred to polyvinylidene fluoride (PVDF) membrane using the Trans Blot Turbo system (Bio-Rad, Hercules, CA, USA). After blocking with 10% skim milk (Becton Dickinson, Franklin Lakes, NJ, USA), the membrane was incubated with primary antibody overnight at 4 °C, and then incubated with 1:5000 dilution of HRP-conjugated secondary antibody for 2 h at room temperature. Chemiluminescence was detected by Chemi-Lumi One Super (Nacalai Tesque, Kyoto, Japan) using the Image Quant LAS 4000 mini (GE Healthcare, Chicago, IL, USA).
To analyze the mobility-shift detection of phosphorylated proteins, we performed Phos-tag SDS-PAGE using Super Sep Phos-tag gels (Fujifilm Wako, Osaka, Japan). The antibodies used in this study are listed in Table S2.

Fertility analysis

Sexually mature control (heterozygous mutant or wild-type) or KO male mice were individually housed with three 6-week-old female B6D2F1 mice for 8 weeks. Male mice were removed after an 8-week mating period and females were maintained for an additional 3 weeks to count the final offspring. The number of pups and copulation plugs were counted every weekday morning.

Sperm analysis

Three KO male mice and age-matched controls were used in this study. To observe sperm morphology, cauda epididymal spermatozoa were suspended in the TYH medium (3). A sperm suspension was placed on a MA coated glass slide (Matsunami Glass, Osaka, Japan), coverslipped (Matsunami Glass) and observed using an Olympus BX53 microscope (Olympus, Tokyo, Japan).
Sperm motility of cauda epididymal spermatozoa suspended in TYH medium were measured using the CEROS II sperm analysis system (software version 1.5; Hamilton Thorne Biosciences, Beverly, MA, USA) after 10 min and 2 hr of incubation.
Propidium Iodide (PI) staining was performed to visually check the viability of spermatozoa. Spermatozoa were incubated with 2.0 μg/mL Hoechst 33342 and 1.0 μg/mL PI in TYH medium and observed using a BX53 microscope or a Nikon Eclipse Ti microscope connected to a C2 confocal module (Nikon, Tokyo, Japan).

In vitro fertilization

In vitro fertilization was performed as previously described (2), using control and KO male mice. Collected spermatozoa were capacitated in vitro for 2 h in TYH medium at 37°C in a humidified atmosphere of 5% CO_2 in air. B6D2F1 female mice were superovulated, and the cumulus-intact oocytes were collected from their oviduct 13-14 h after hCG injection, followed by incubation in TYH medium. Cumulus-intact, cumulus-free, or zona-free oocytes were incubated with 2.0×10^5 sperm/mL for 6 h. After 6 h co-incubation, formation of pronuclei were observed using a differential interference contrast (DIC) microscope (Olympus IX73). Frequencies of 2-cell embryos were observed 24 h after insemination using the same DIC microscope. Fertilization rates of both cumulus-intact and -free oocytes were examined by counting the number of 2-cell embryos, and that of zona-free oocytes was examined by counting the number of embryos with pronuclei.

Intracytoplasmic sperm injection (ICSI)

ICSI was performed as previously described (4). Whole epididymal spermatozoa were injected into mature mouse oocytes using a piezo manipulator (PrimeTech, Ibaraki, Japan). The following day, two-cell embryos obtained by ICSI and WT embryos obtained by ICR x ICR mating were transferred to pseudopregnant females. Genotyping was conducted one week after the birth.

Periodic acid–Schiff (PAS) and toluidine blue (TB) staining of testis

Testes of heterozygous male and KO male mice were dissected after euthanasia. Testis weights were measured, after which the testes were fixed in Bouin's fluid (Polysciences, Inc., Warrington, PA, USA) and embedded in paraffin. Sections were cut at 5 μm by microtome (HM325, Microm, Walldorf, Germany), fixed on glass slides.

For PAS staining, sections were deparaffined in xylene, rehydrated, treated with 1% periodic acid for 10 min, and Schiff's reagent (Fujifilm Wako) for 20 min. Sections were stained with Mayer's hematoxylin solution (Fujifilm Wako), coverslipped and observed using a BX53 microscope.

For TB staining, sections were deparaffined in xylene, rehydrated, treated with 0.5% toluidine blue O reagent with 1% acetic acid for 5 min. Sections were washed with 100% ethanol, coverslipped and observed using a BX53 microscope.

Immunoprecipitation

Cell lysates from mouse testis were obtained using a T-PER, following the manufacturer's instructions. Solubilized proteins were mixed with Dynabeads Protein G (ThermoFisher Scientific)-conjugated antibody. The immune complexes were incubated for 1 h at 4°C and co-immunoprecipitated (co-IP) products were eluted with SDS-sample buffer and denatured for 10 min at 70 °C. The antibodies used in this study are listed in Table S2.

MS analysis using testicular lysate

Testicular lysates were extracted using a T-PER. Immunoprecipitation was performed as described above using an anti-TSKS antibody. Obtained immunoprecipitated proteins were subjected to MS analysis as previously described (5). The proteins were reduced, alkylated and digested. The resultant peptides were subjected to nanocapillary reversed-phase LC-MS/MS analysis using a C18 column (25 cm × 75 um, 1.6 μm; IonOpticks, Victoria, Australia) on a nanoLC system (Bruker Daltoniks, Bremen, Germany) connected to a timsTOF Pro mass spectrometer (Bruker Daltoniks) and a modified nano-electrospray ion source (CaptiveSpray; Bruker Daltoniks). The resulting data was processed using DataAnalysis version 5.1 (Bruker Daltoniks), and proteins were identified using MASCOT version 2.7.0 (Matrix Science, London, UK) against the Swiss-Prot database. Quantitative value and fold exchange were calculated by Scaffold5 (Proteome Software, Portland, OR, USA) for MS/MS-based proteomic studies

Construction of expression plasmids

cDNAs encoding *Tsks* (XM_006540796.3), *Tssk1* (NM_009435.2), *Tssk2* (NM_009436.2), *PPP1cc2* (XM_006530200) were amplified from mouse testis (C57BL/6N). *Tsks* cDNA was cloned into 1D4-tagged (C terminus) pCAG vectors that contain the CAG promoter and a rabbit globin poly (A) signal (6). *Tssk1* cDNAs, *Tssk2* cDNA, *Ppp1cc2* were cloned into the pCAG vector with Myc tag (N terminus), 3xFLAG (C terminus) or HA tag (N-terminus), respectively. Primers used to construct these plasmids are listed in Table S1.

SI Figures

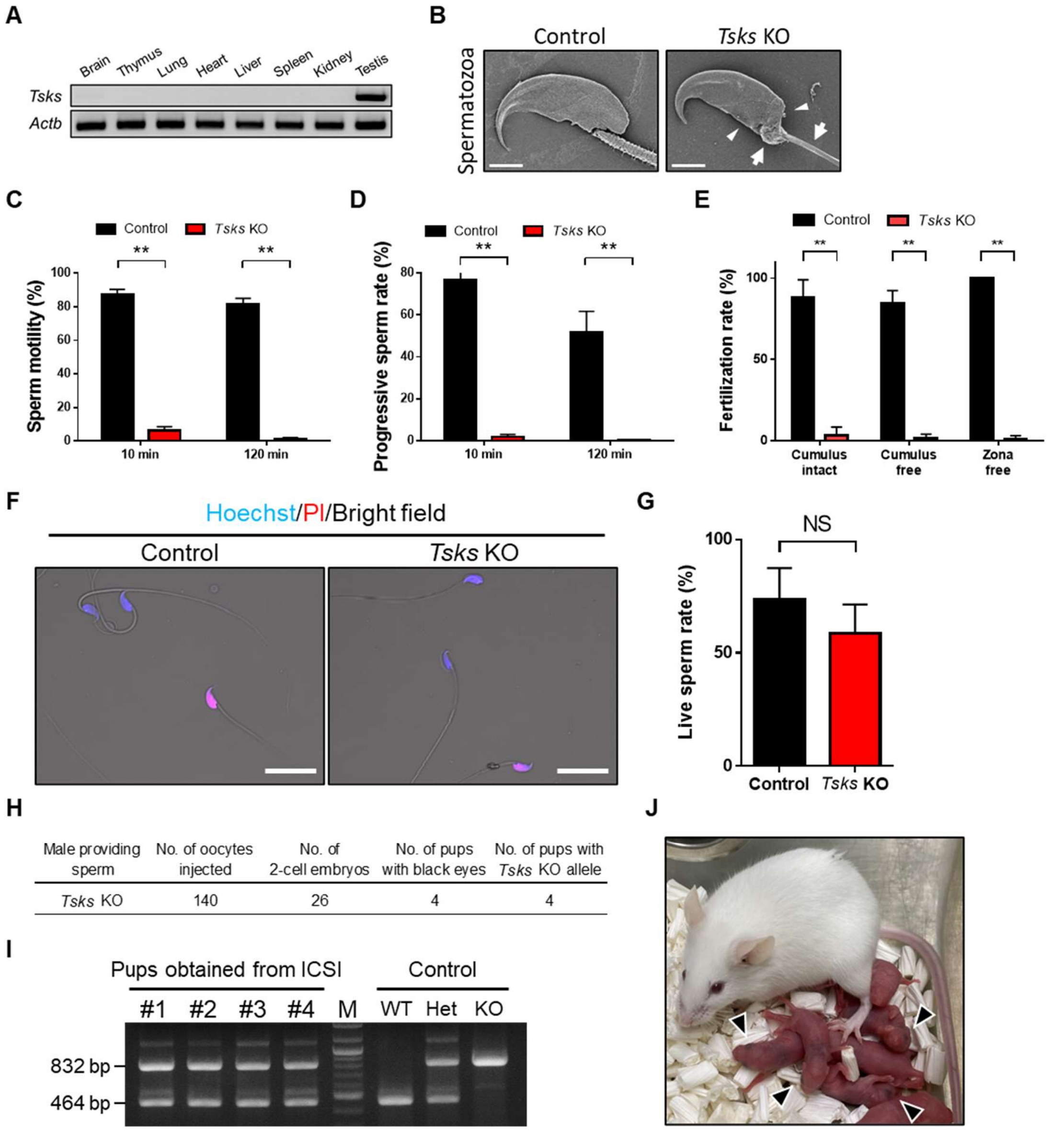

Fig. S1. *Tsks*-deleted spermatozoa show low motility, but normal pups are obtained with ICSI

(A) The expression of mouse *Tsks* was examined by RT-PCR using RNA isolated from various organs. *Tsks* shows testis-enriched expression. *Actb* was used as a loading control. (B) SEM images of sperm heads collected from the cauda epididymis of control and *Tsks* KO mice. Arrows and arrowheads indicate abnormal mitochondrial alignment and abnormal sperm head shape, respectively. Scale bars: 2.0 μm. (C-D) Sperm motility (C) and progressive sperm rate (D) from control and *Tsks* KO mice (**P < 0.01, Student's t test; error bars represent SD, *N* = 3). (E) Fertilization rate of IVF using control and *Tsks* KO spermatozoa. Three types of oocytes (cumulus-intact, cumulus-free, and zona-free) were used for IVF (**P < 0.01, Student's t test; error bars represent SD, *N* = 3). (F) Spermatozoa collected from control and *Tsks* KO mice were stained with Hoechst 33342 (blue) and propidium iodide (red) to check live/dead ratio. Scale bars: 20 μm. (G) Graph indicates live sperm rates in control and *Tsks* KO spermatozoa (*N* = 3). There

was no significant difference between control and *Tsks* KO (P = 0.25). (H) Twenty-six (18.6%) of the 140 oocytes injected with the spermatozoa from *Tsks* KO male mice developed to the two-cell stage, and four pups were obtained after embryo transfer. (I) Genotyping of obtained pups. All four pups were derived from *Tsks* KO spermatozoa. (J) Pups with black eyes were derived from *Tsks* KO spermatozoa (arrowheads). Other pups were derived from WT embryos that were transplanted at the same time as embryos obtained by ICSI.

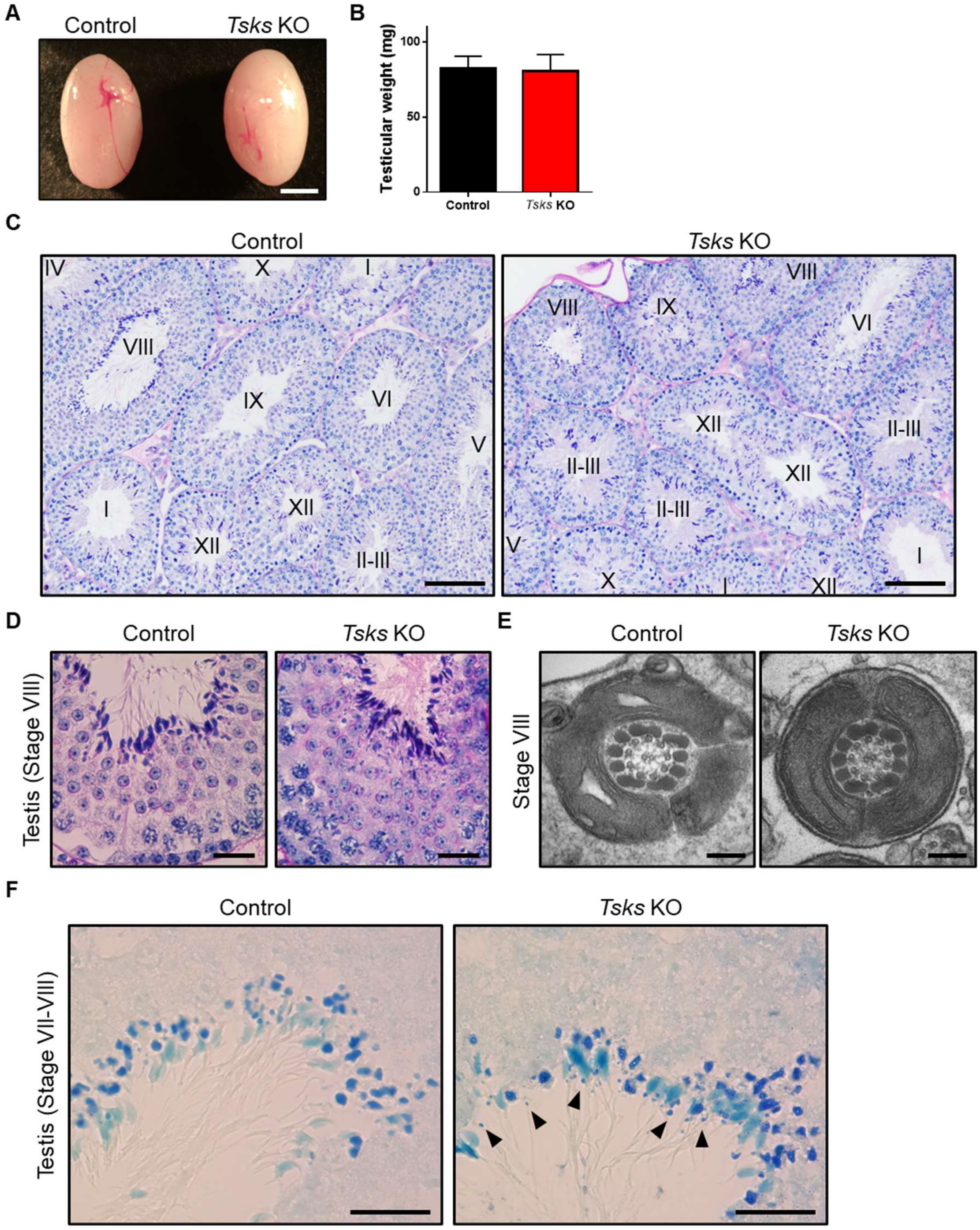

Fig. S2. *Tsks*-deleted male mice show abnormal spermatogenesis

(A) Gross morphology of control and *Tsks* KO testes. Scale bars: 2.0 mm. (B) Testis weight of control and *Tsks* KO mice (N = 5). Average weight of testis was control = 82.1 ± 7.8 mg; *Tsks* KO = 80.5 ± 10.4 mg. P = 0.7186. (C) PAS staining of testis samples from control and *Tsks* KO male mice. Sperm heads were observed in stage IX seminiferous tubule in *Tsks* KO testis. Scale bars: 100 µm. (D) PAS staining of stage VIII seminiferous tubules in the testis was observed at high magnification. In stage VIII seminiferous tubules, *Tsks* KO testis appears to be crowded with a large number of sperm heads present in the lumen. In contrast, spermatozoa in control testis are

in a row in the lumen of the testis. Scale bars: 20 μm. (E) Ultrastructural images of flagella in the midpiece of step 16 spermatids. No abnormalities were observed. Scale bars: 200 nm. (F) Toluidine blue staining for control and *Tsks* KO testis section. Dark blue-stained area shows residual bodies or excess residual cytoplasm (arrowheads). Light blue (known as metachromasia) area shows sperm heads. Scale bars: 20 μm.

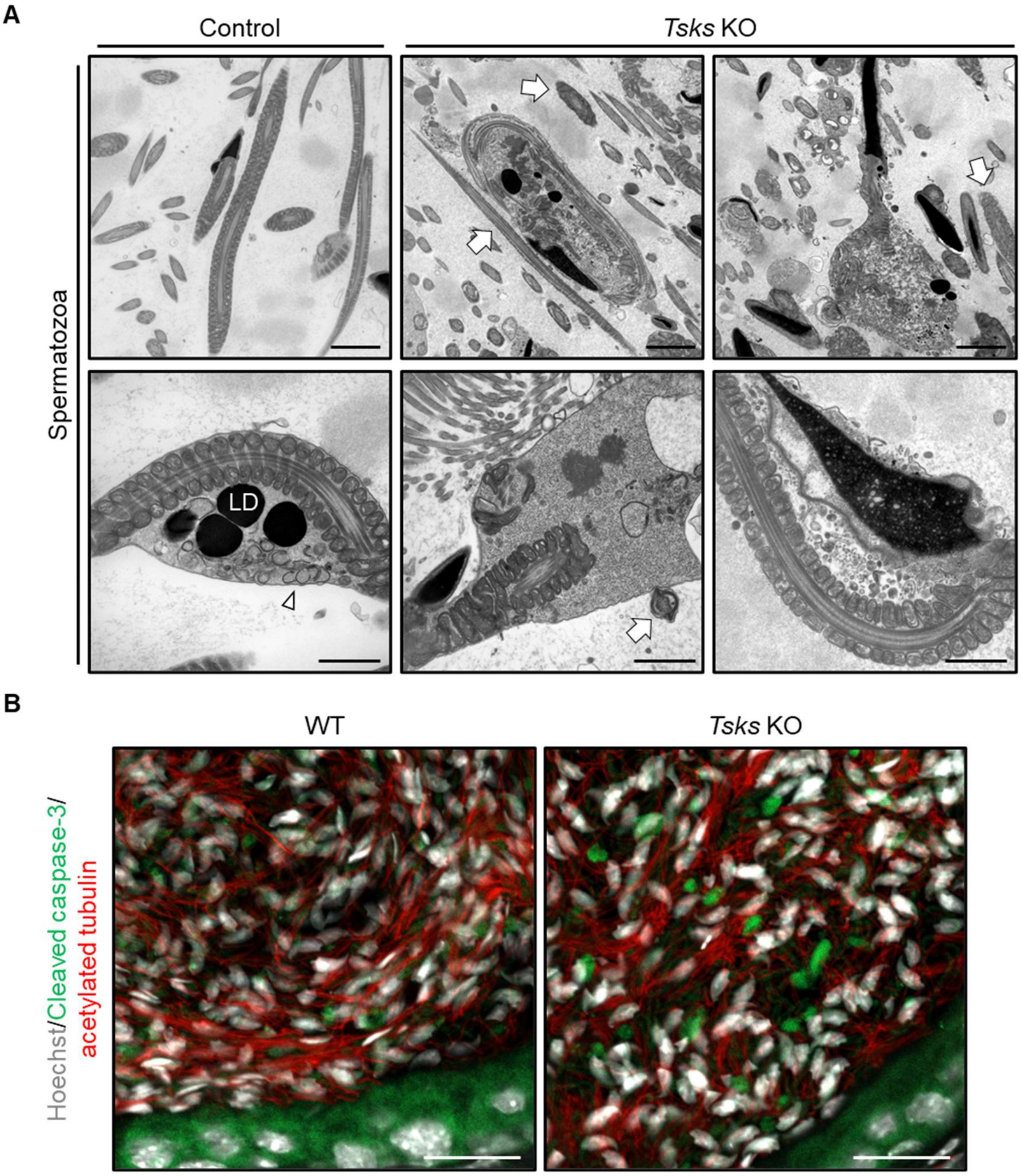

Fig. S3. *Tsks*-deleted spermatozoa with excess residual cytoplasm exhibit an apoptotic response

(A) Ultrastructural images of spermatozoa in cauda epididymis. Cytoplasmic droplet in WT spermatozoon contains lipid droplets (LD) and flattened saccular elements (arrowhead). *Tsks* KO spermatozoa with ERC have incomplete membrane and/or substances with high electron density. As indicated by the arrows, parts without ERC have few abnormalities. Scale bars: 2 µm (upper panels), 1 µm (lower panels). (B) Immunofluorescence staining of the cauda epididymis of WT and *Tsks* KO mice with antibodies to cleaved Caspase-3 (green). Hoechst 33342 (white) and acetylated tubulin (red) were used to visualize the nuclei and tubulin, respectively. Scale bars: 20 µm.

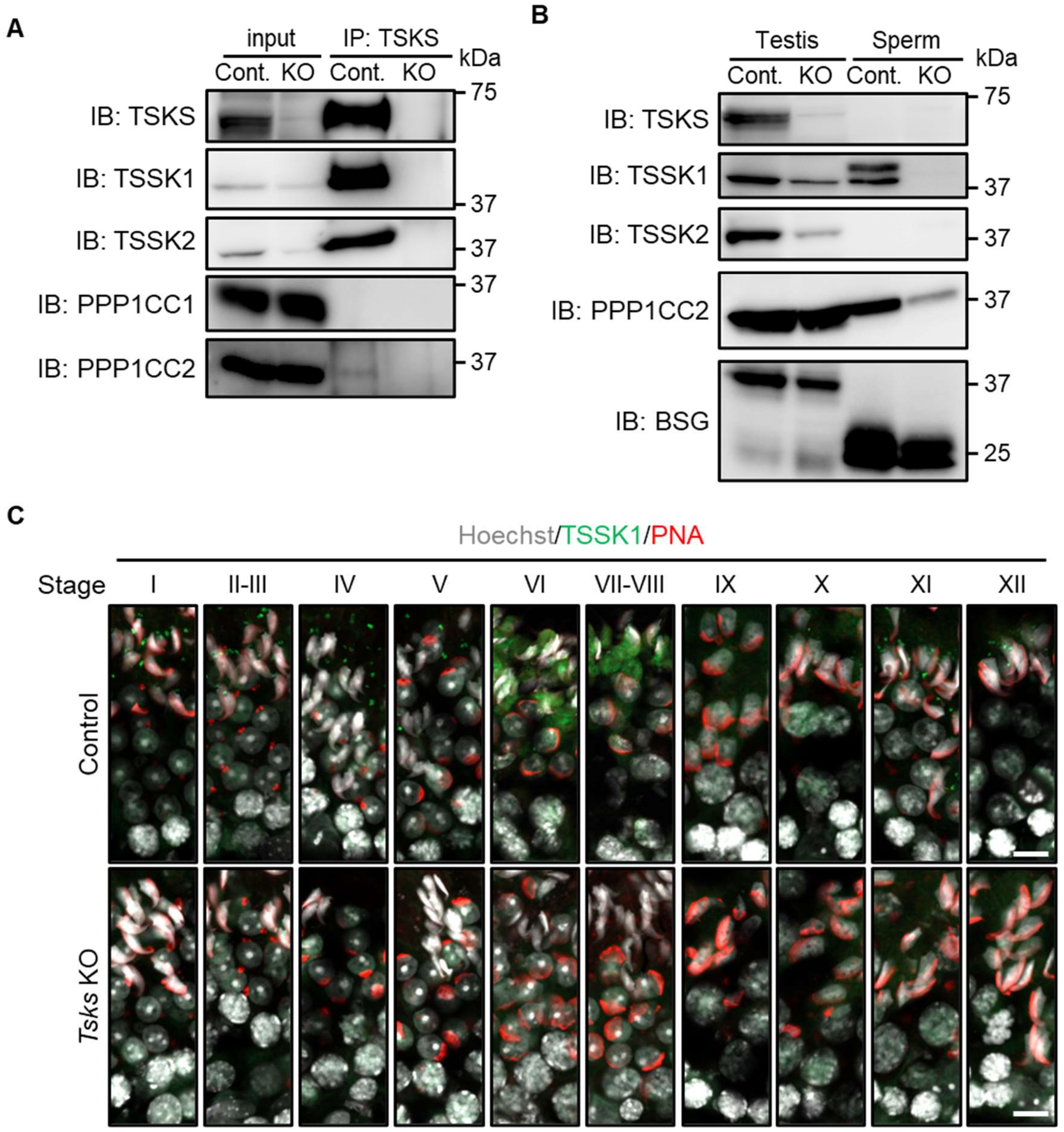
A
input
IP: TSKS
Cont.
KO
Cont.
KO
kDa
IB: TSKS
IB: TSSK1
IB: TSSK2
IB: PPP1CC1
IB: PPP1CC2
75
37
37
37
37
B
Testis
Sperm
Cont.
KO
Cont.
KO
kDa
IB: TSKS
IB: TSSK1
IB: TSSK2
IB: PPP1CC2
IB: BSG
75
37
37
37
37
25
C
Hoechst/TSSK1/PNA
Stage
I
II-III
IV
V
VI
VII-VIII
IX
X
XI
XII
Control
Tsks KO

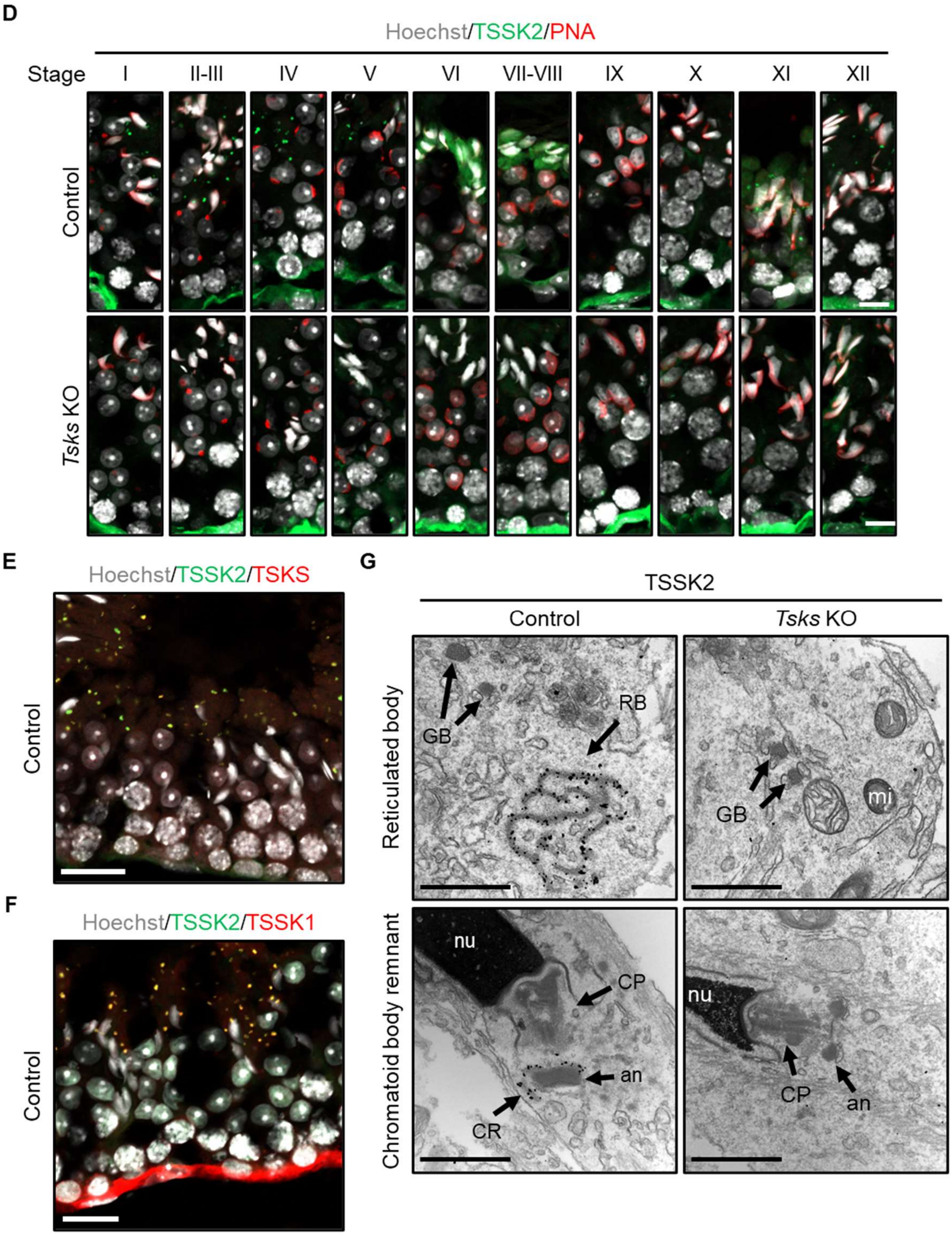

Fig. S4. Both TSSK1 and TSSK2 co-localize with TSKS
(A) Co-IP followed by WB analysis were performed using lysates collected from control or *Tsks* KO mice testis. Immunoprecipitated proteins by anti-TSKS antibody were analyzed by WB analysis using anti-TSKS, TSSK1, TSSK2, PPP1CC1, and PPP1CC2 antibodies. (B) WB analysis using lysates prepared from the testis and mature spermatozoa from control and *Tsks* KO mice. BSG was used as a loading control. (C-D) Immunofluorescence analysis of stage I-XII

seminiferous tubules in the testis of control and *Tsks* KO mice. Spermatogenic stages were identified by the morphology of the nucleus and acrosome stained with Hoechst 33342 (white) and PNA-lectin (red), respectively. TSSK1 (C) and TSSK2 (D) were detected with antibodies to TSSK1 and TSSK2, respectively. Both TSSK1 and TSSK2 were expressed with strong dot signals during step 11 to 15 spermatids at stage V seminiferous tubules in control as well as TSKS. However, both TSSK proteins were expressed whole cytoplasm in elongated spermatids after TSKS was disappeared (stage VI to VIII seminiferous tubules). Scale bars: 10 μm. (E-F) Immunofluorescence staining of the testis of WT mice with antibodies to TSSK2 (green) and TSKS (E) or TSSK1 (F). Nuclei were stained with Hoechst 33342 (white). Scale bars: 20 μm. (G) Detection of immunolabeled TSSK2 in testis by TEM using anti-TSSK2 antibody incubated with 1.4-nm gold-particle–conjugated secondary antibody. TSSK2 localizes on two different nuage; RB and CR just like TSKS. Scale bars: 1 μm. RB, reticulated body; CR, chromatoid body remnant; GB, granulated body; CP, connecting piece; mi, mitochondria; nu, nuclear; an, annulus.

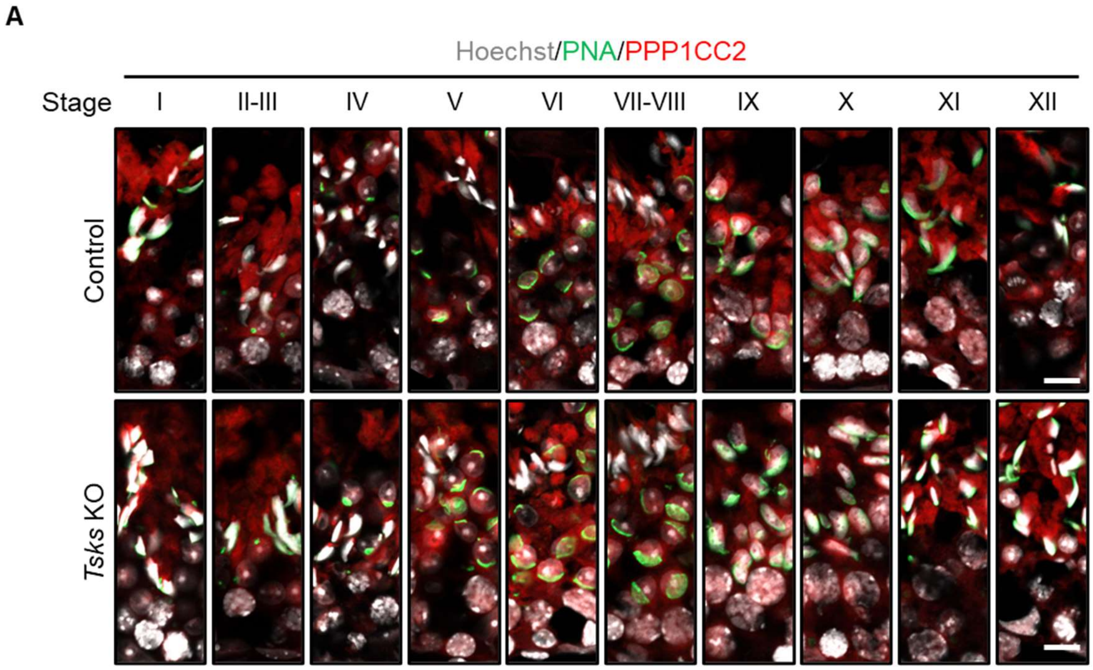

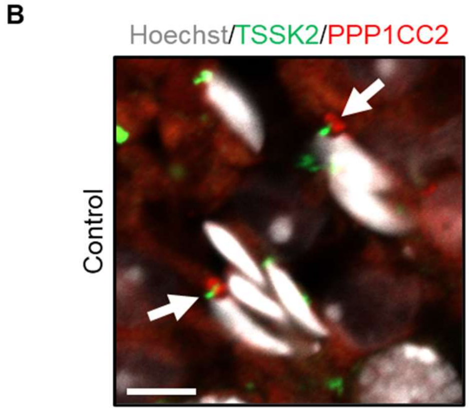

Fig. S5. PPP1CC2 localizes near TSKS complex
(A) Immunofluorescence analysis of stage I-XII seminiferous tubules in the testis of control and *Tsks* KO mice. Spermatogenic stages were identified by the morphology of the nucleus and acrosome stained with Hoechst 33342 (white) and PNA-lectin (green), respectively. PPP1CC2 was detected with antibodies to PPP1CC2 (red). PPP1CC2 expresses in the cytoplasm of late spermatocytes and whole spermatids of control and *Tsks* KO testis. Scale bars: 10 μm. (B) Enlarged image of stage II-III seminiferous tubule in the testis of WT mouse. PPP1CC2 (red) does not co-localize with TSSK2 (green), but prominent expression of PPP1CC2 were observed near TSSK2 signals (arrows). Nuclei were stained with Hoechst 33342 (white). Scale bar: 5 μm.

A

#	Protein name	Quantitative value: Control	Quantitative value: *Tsks* KO	Ratio
1	TSKS	59	0	-
2	TSSK1	18	0	-
3	HSPA8	15	0	-
4	TSSK2	9	0	-
5	PPP1CC	7	0	-
6	ODF1	7	0	-
7	CCDC91	7	0	-
8	UHRF1BP1L	7	0	-
9	ACTB	6	0	-
10	HSPA1L	5	0	-
11	HSPA5	12	1	12.0
12	HSPA2	23	3	7.7
13	KRT5	6	1	6.0
14	EFHD1	6	1	6.0

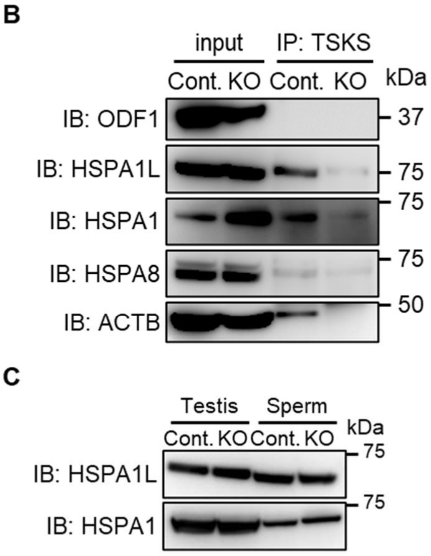

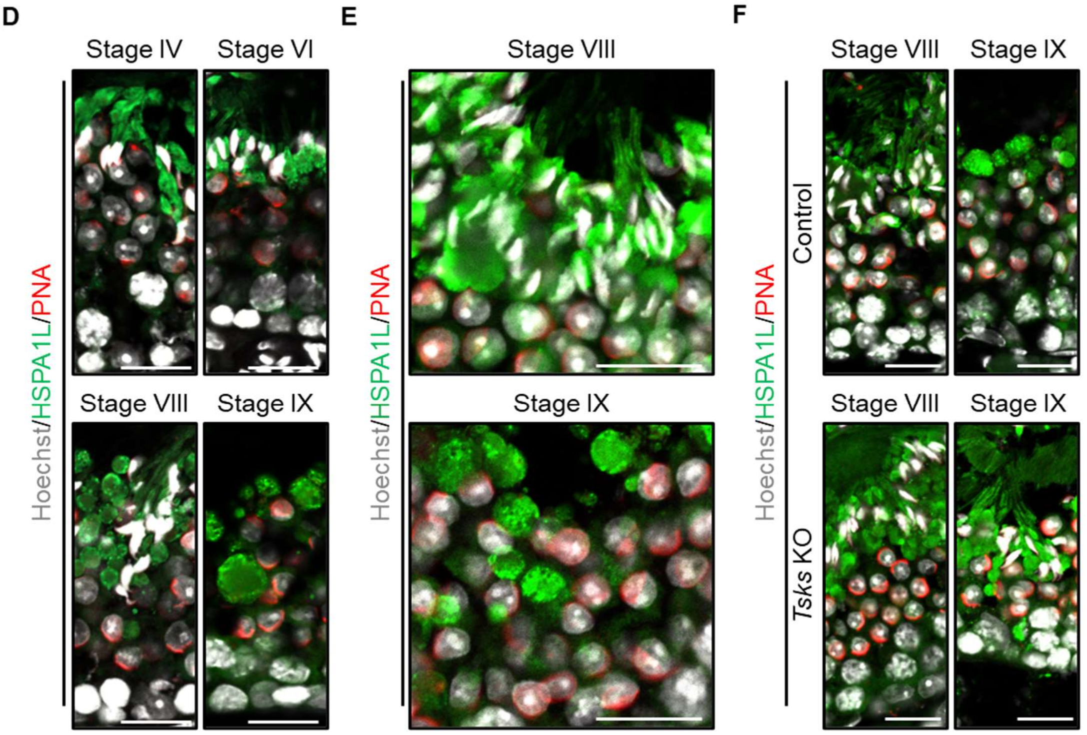

Fig. S6. TSKS interacts with HSPA1L, which localizes to the cytoplasmic lobe and residual body

(A) List of identified proteins by MS analysis in vivo. Proteins were extracted from control or *Tsks* KO testes. The proteins were immunoprecipitated using anti-TSKS antibody and identified by MS analysis. This list shows proteins either identified only in control (quantitative value =>5.0) or highly identified in control (ratio =>6.0 as compared with *Tsks* KO) are listed. (B) Co-IP followed by WB analysis were performed using lysates collected from control or *Tsks* KO mouse testes. Immunoprecipitated proteins by anti-TSKS antibody were analyzed by WB using anti-ODF1, HSPA1L, HSPA1, HSPA8, and ACTB antibodies. (C) Western blot analysis using lysates prepared from the testis and mature spermatozoa from control and *Tsks* KO mice. (D-F) Immunostaining of HSPA1L (green) in WT or *Tsks* KO mouse testis. Hoechst 33342 (white) and

PNA-lectin (red) were used to visualize the nuclei and acrosome, respectively. Scale bars: 20 μm. (D) Immunostaining of HSPA1L in WT mouse testis (stage IV, VI, VIII, IX seminiferous tubules). HSPA1L is located in the cytoplasm in the lumen of seminiferous tubules at stage IV of the spermatogenic cycle. Then, the location of HSPA1L is changed near the sperm head (stage VI). Before spermiation, HSPA1L localizes in the cytoplasm including the cytoplasmic lobe and midpiece (stage VIII), and HSPA1L is observed in the residual bodies after sperm release (stage IX). (E) Immunostaining of HSPA1L in WT mouse testis before and after sperm release with high magnification. (F) Immunostaining of HSPA1L in control and *Tsks* KO mouse testis. HSPA1L in *Tsks* KO testis at stage VIII of the spermatogenic cycle is expressed broadly both in the cytoplasm lobe and cytoplasm in the seminiferous tubule lumen. HSPA1L expression in the sperm midpiece and residual bodies was observed in *Tsks* KO testis at Stage IX seminiferous tubules.

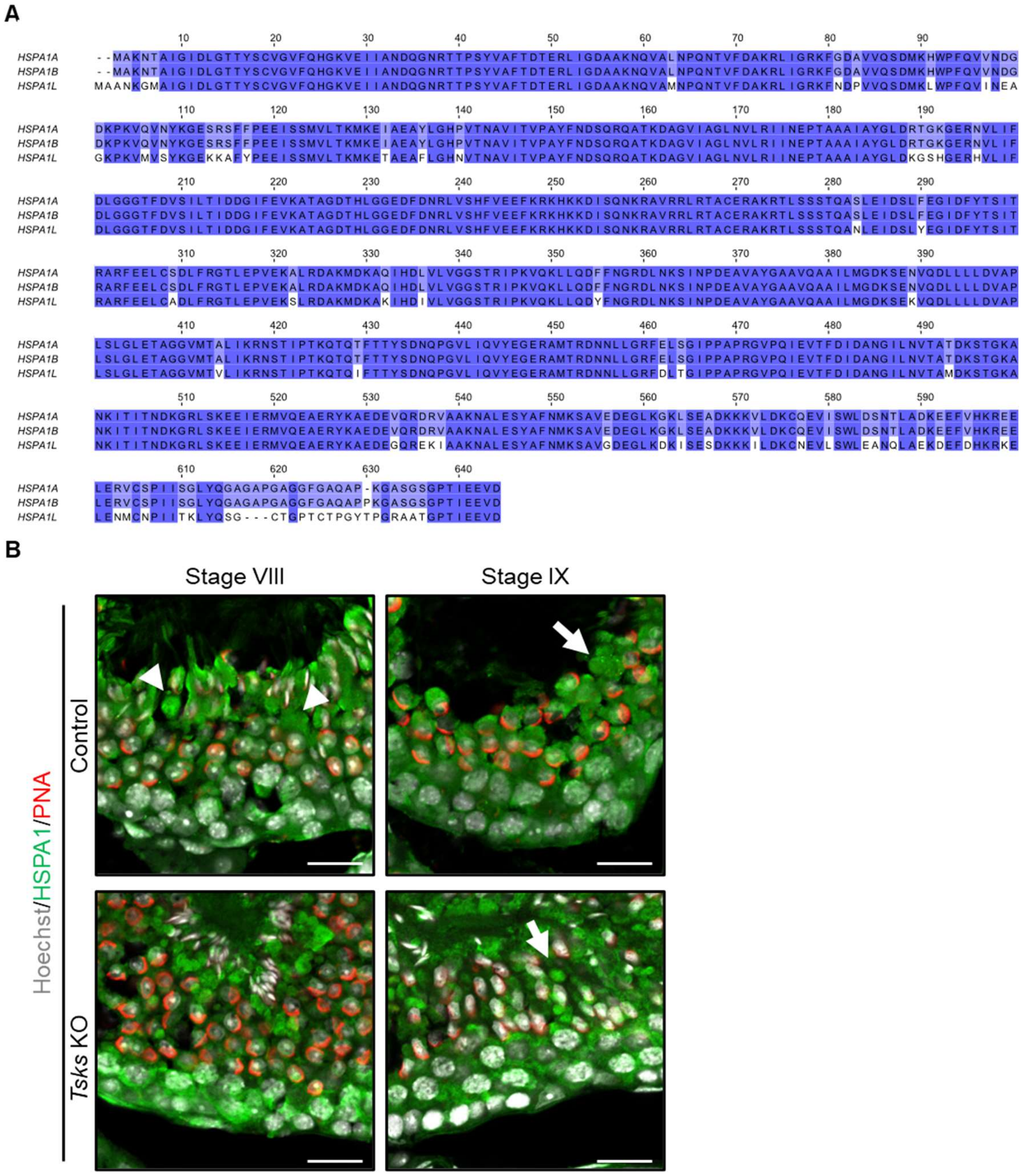

Fig. S7. The paralog of HSPA1L, HSPA1, localizes to the cytoplasmic lobe and residual body

(A) Sequence alignment of HSPA1A, HSPA1B and HSPA1L proteins from mouse. (B) Immunostaining of HSPA1 in control and *Tsks* KO mouse testis at stage VIII and IX seminiferous tubules. HSPA1 was expressed throughout the testis including the cytoplasmic lobe (arrowheads) and residual body (arrows). Scale bars: 20 µm.

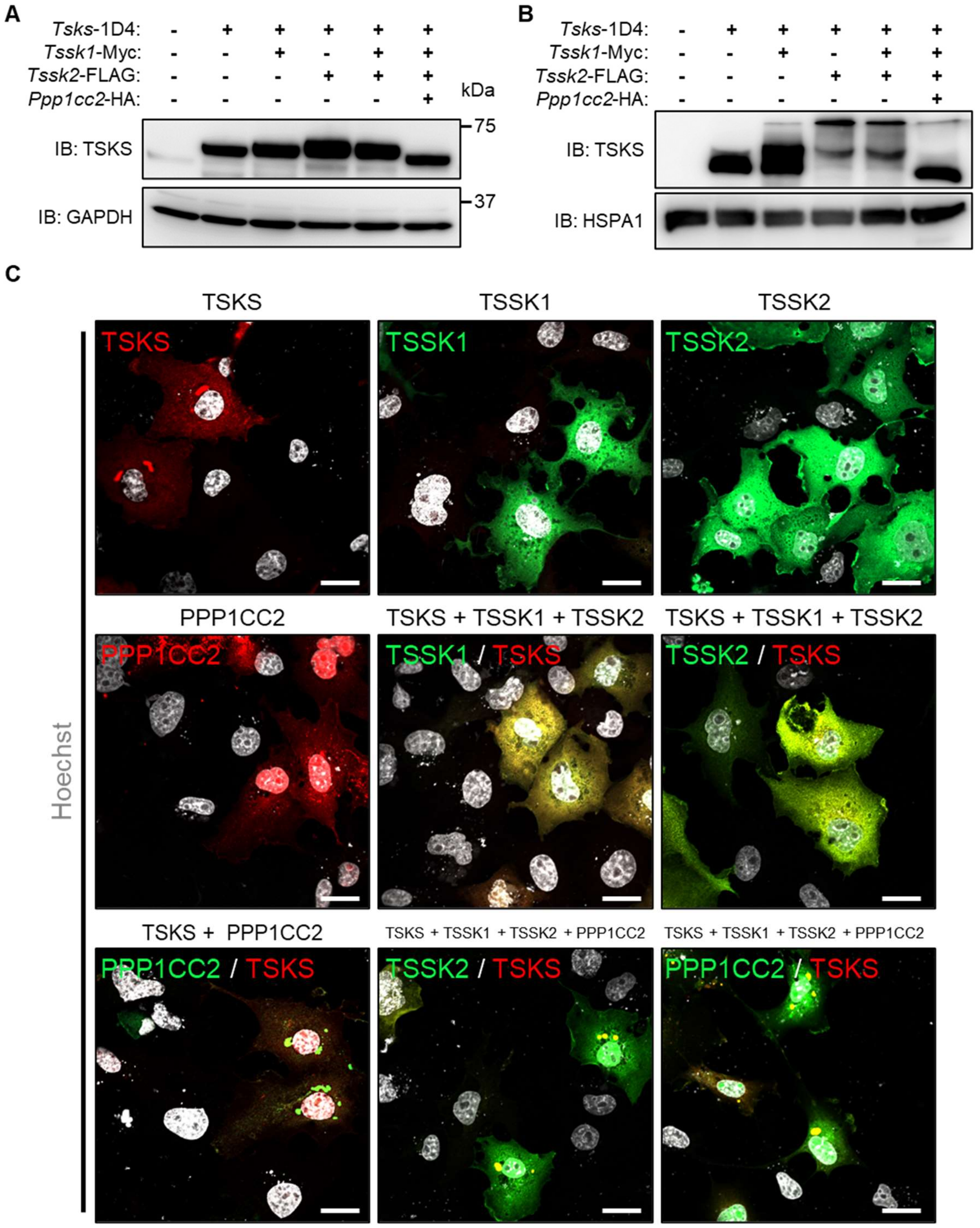

Fig. S8. Phosphorylation of TSKS inhibits nuage formation in vitro
(A-B) HEK293T cells were transfected with plasmids encoding 1D4-tagged TSKS and/or its kinase and phosphatase. (A) Cell lysates were subjected to general SDS-PAGE and analyzed by immunoblot using an anti-TSKS antibody. GAPDH was used as a loading control. (B) Cell lysates were subjected to Phos-tag SDS-PAGE assay with TSKS antibody. Shift bands (top and middle) represented phosphorylated TSKS; bottom bands represented non-phosphorylated TSKS. Both TSSK1 and TSSK2 phosphorylate TSKS, but PPP1CC2 dephosphorylates TSKS even when TSSK1 and TSSK2 are present. HSPA1 was used as a loading control, which does not show any

mobility shift. (C) COS-7 cells transiently expressing TSKS, TSSK1, TSSK2, or PPP1CC2. Hoechst 33342 (white) was used to visualize the nuclei. While single expression of TSSK1, TSSK2 and PPP1CC2 does not induce droplets, kinase and phosphatase proteins co-localize with TSKS when TSKS is dephosphorylated. Scale bars: 20 µm.

SI Tables

Table S1. Primer list

Method	Target	Primer sequence	Annealing temperature (°C)	Cycles
RT-PCR	*Actb*	Fw: CATCCGTAAAGACCTCTATGCCAAC<br>Rv: ATGGAGCCACCGATCCACA	60	35
	Tsks	Fw: GTCCTGACCCAGGAGAACTG<br>Rv: ACTGCTGACCTCTCCACCAT	60	35
Genotyping	*Tsks* WT allele	Fw: TACCCCTCTCTGTGACCCAG<br>Rv: CCCAACCCTCTCCTGATTCC	65	40
	Tsks KO allele	Fw: TACCCCTCTCTGTGACCCAG<br>Rv: CACCTGGCCTTATGCAGTGAG	65	40
Cloning	*Tsks*	Fw: GTCGACGCCGCCATGGCAAGCGTGGTGGTGAA<br>Rv: GAATTCTTGTTCAGAGCCTGGACTCC	65	40
	Tssk1	Fw: GAATTCATGGATGACGCTGCCGTCCT<br>Rv: GCGGCCGCCTAAGTATGTGTCTCTGAAGGCTG	65	40
	Tssk2	Fw: GAATTCGCCGCCATGGACGATGCGGCGGTCCT<br>Rv: GTCGACGGTACTTGCTTTCTCCACCTCAGC	65	40
	PPP1CC2	Fw: GAATTCATGGCGGATATCGACAAACT<br>Rv: GTCGACTCACTCGTATAGGACAGTGTTG	65	40

Table S2. Antibody list

Immunoblot analysis

Antibody	Clone No.	Host species	Catalog No.	Company	Dilution
anti-1D4	polyclonal	Rabbit	-	In house	1:1000
anti-ACTB	AC-15	Mouse	ab6276	abcam	1:500
anti-BSG (EMMPRIN)	B-5	Mouse	sc-46700	Santa Cruz	1:2000
anti-GAPDH	14C10	Rabbit	#2118	Cell Signaling	1:1000
anti-HSPA1 (HSP70)	polyclonal	Rabbit	10995-1-AP	Proteintech	1:5000
anti-HSPA1L	polyclonal	Rabbit	NBP1-92012	Novus	1:1000
anti-HSPA8 (Hsc70)	polyclonal	Rabbit	10654-1-AP	Proteintech	1:5000
anti-ODF1	E-11	Mouse	sc-390152	Santa Cruz	1:500
anti-PPP1CC1 (PP1γ1)	polyclonal	Rabbit	Gift from Srinivasan Vijayaraghavan		1:5000
anti-PPP1CC2 (PP1γ2)	polyclonal	Rabbit	Gift from Srinivasan Vijayaraghavan		1:2000
anti-TSKS	polyclonal	Rabbit	-	In house	1:500
anti-TSSK1	polyclonal	Rabbit	-	In house	1:2000
anti-TSSK2	1E12	Mouse	H00023617-M01	Avnova	1:500

Immunofluorescence analysis

Antibody	Clone No.	Host species	Catalog No.	Company	Dilution
anti-1D4 tag	polyclonal	Rabbit	-	In house	1:1000
anti-Acetylated tubulin	6-11B-1	Mouse	T7451	Merck	1:500
anti-Cleaved Caspase-3	5A1E	Rabbit	#9664	Cell Signaling	1:1000
anti-FLAG tag	M2	Mouse	F1804	Merck	1:1000
anti-HA tag	TANA2	Mouse	M180-3	MBL	1:500
anti-HSPA1 (HSP70)	polyclonal	Rabbit	10995-1-AP	Proteintech	1:500
anti-HSPA1L	polyclonal	Rabbit	NBP1-92012	Novus	1:500
anti-Myc tag	9B11	Mouse	#2276	Cell Signaling	1:500
anti-PPP1CC2 (PP1γ2)	polyclonal	Rabbit	Gift from Srinivasan Vijayaraghavan		1:500
anti-TSKS	polyclonal	Rabbit	-	In house	1:500
anti-TSSK1	polyclonal	Rabbit	-	In house	1:1000
anti-TSSK2	1E12	Mouse	H00023617-M01	Avnova	1:250

Immunoelectron microscopy

Antibody	Clone No.	Host species	Catalog No.	Company	Dilution
anti-TSKS	polyclonal	Rabbit	-	In house	1:150
anti-TSSK2	1E12	Mouse	H00023617-M01	Avnova	1:150

Immunoprecipitation

Antibody	Clone No.	Host species	Catalog No.	Company
anti-TSKS	polyclonal	Rabbit	-	In house
